# Using Qualitative and Quantitative Methods to Choose a Habitat Quality Metric for Air Pollution Policy Evaluation

**DOI:** 10.1371/journal.pone.0161085

**Published:** 2016-08-24

**Authors:** Edwin C. Rowe, Adriana E. S. Ford, Simon M. Smart, Peter A. Henrys, Mike R. Ashmore

**Affiliations:** 1 Centre for Ecology and Hydrology, Bangor, Gwynedd, United Kingdom; 2 Environment Department, University of York, North Yorkshire, United Kingdom; 3 Faculty of Architecture, Computing & Humanities, University of Greenwich, London, United Kingdom; 4 Centre for Ecology and Hydrology, Lancaster, Lancashire, United Kingdom; 5 Stockholm Environment Institute, University of York, York, North Yorkshire, United Kingdom; York University, CANADA

## Abstract

Atmospheric nitrogen (N) deposition has had detrimental effects on species composition in a range of sensitive habitats, although N deposition can also increase agricultural productivity and carbon storage, and favours a few species considered of importance for conservation. Conservation targets are multiple, and increasingly incorporate services derived from nature as well as concepts of intrinsic value. Priorities vary. How then should changes in a set of species caused by drivers such as N deposition be assessed? We used a novel combination of qualitative semi-structured interviews and quantitative ranking to elucidate the views of conservation professionals specialising in grasslands, heathlands and mires. Although conservation management goals are varied, terrestrial habitat quality is mainly assessed by these specialists on the basis of plant species, since these are readily observed. The presence and abundance of plant species that are scarce, or have important functional roles, emerged as important criteria for judging overall habitat quality. However, species defined as ‘positive indicator-species’ (not particularly scarce, but distinctive for the habitat) were considered particularly important. Scarce species are by definition not always found, and the presence of functionally important species is not a sufficient indicator of site quality. Habitat quality as assessed by the key informants was rank-correlated with the number of positive indicator-species present at a site for seven of the nine habitat classes assessed. Other metrics such as species-richness or a metric of scarcity were inconsistently or not correlated with the specialists’ assessments. We recommend that metrics of habitat quality used to assess N pollution impacts are based on the occurrence of, or habitat-suitability for, distinctive species. Metrics of this type are likely to be widely applicable for assessing habitat change in response to different drivers. The novel combined qualitative and quantitative approach taken to elucidate the priorities of conservation professionals could be usefully applied in other contexts.

## Introduction

Atmospheric nitrogen (N) pollution is having severe impacts on human health [1] and is a worldwide threat to biodiversity [2,3]. Nitrogen changes the competitive balance among plant species, and those that are negatively affected by N tend to have greater threat status [4]. Nitrogen pollution also influences a variety of other taxa (e.g. [5]). However, N pollution can favour species that indicate good site condition such as certain *Sphagnum* mosses [6], and species which are important components of protected habitats [7,8]. Species richness may increase despite long-term N application when species that are adapted for nutrient-rich habitats invade [9]. Despite these ostensibly beneficial effects it is widely recognised that atmospheric N pollution has mainly negative impacts on biodiversity [10–12]. Policy development in this area makes extensive use of simulations of the ecological impacts of N pollution scenarios [13,14]. Models are available which simulate aspects such as habitat-suitability for phyto-sociological communities [15], proportional cover of plant species [16], or habitat-suitability for plant and lichen species [17–19], and these models are often used to explore air pollution scenarios, e.g. at the annual workshop of the Co-ordination Centre for Effects (CCE) of the Convention on Long Range Transboundary Air Pollution. Progress made in terms of predicting ecological responses has however outstripped the capacity to interpret these changes in terms of biodiversity targets. In the current study we consulted habitat specialists using qualitative and quantitative methods to determine an appropriate basis for a habitat quality metric for use in this context.

Biodiversity is a complex and much-debated concept, and it is often argued that it needs to be represented using many dimensions (e.g. [20–22]). A project designed to streamline biodiversity indicators still resulted in a short-list of 26 indicators [23]. However, air pollution policy makes extensive use of the concept of critical load [24], which relies on assessment of whether a habitat is damaged or not, and thus requires definition of a single summary metric of habitat quality (HQ). A one-dimensional HQ metric is also needed if biodiversity impacts are to be included in integrated assessments of air pollution effects across sectors [25]. In this study the term ‘habitat’ is used in the sense of a biotope, i.e. an area with environmental conditions that support a particular biotic assemblage, rather than in the sense of the environment of a particular species. The term ‘quality’ is used in the sense of ‘degree of excellence’ [26].

The chance of achieving agreement on a biodiversity metric may be increased by limiting its scope to terrestrial ecosystems. Also, since transitions between major habitat types are more usually mediated by management than pollution, it is often possible to disregard β- and γ-diversity in the context of pollution impacts and to consider the quality of the habitat rather than site-scale or landscape-scale biodiversity. Nevertheless, even for a single habitat many different approaches can be taken to defining biodiversity value, such as species richness, or the presence or abundance of particular taxa. Taxa considered important may be scarce or declining species; those important to the structure, function or integrity of the habitat; and/or those that provide important ecosystem services [27]. Species scarcity can be defined with reference to global, national or local populations. It may be argued that preventing extinction at global scale is of critical importance [28], and this is the basis of the “Red List” [29,30]. However, a local perspective can also be important, in that encountering species that are unusual or rare in a particular area foments an interest in nature conservation and ensures that nature conservation values persist in human society [31]. The presence of competitive, invasive or ubiquitous taxa is often considered to reduce biodiversity, although whether non-native species should necessarily be considered negative is debated [32–34]. Biodiversity can be defined as similarity to a pre-industrial or other reference state, although there is some conflict between the ideal of “naturalness” and the appreciation that some human interventions can increase biodiversity [35,36].

Scientific justifications can be constructed for many of these criteria, but ultimately the balance of considerations used to define HQ depends on the value-system and priorities of whoever is making the judgement [37–40]. This conclusion is rejected by many biodiversity scientists, and earlier versions of this article were indeed rejected by several journals for reasons best summarised by the comment of one reviewer: “the scientific basis of conservation is a rule”. However, it is difficult to see how biodiversity can be assessed in isolation from value judgements [41], and appropriate policy responses are best developed using insights and methods from both natural and social science [42]. The necessity for value judgments cannot be avoided by combining multiple criteria into a single metric, since the weighting of components is inevitably subjective and the combined metric is likely to be rather opaque [43]. The Biodiversity Indicators Partnership recommends discussion of key questions, and the use to which a potential biodiversity indicator will be put, in collaboration with stakeholders [44], and this was the guiding principle for our study.

The aim of the study was to assess quantitative indicators of HQ for their potential for evaluating N pollution impacts over a range of habitats. Our intention was to identify a general metric of habitat quality, not to assess whether this metric explicitly related to N deposition. Although the context of the study was an assessment of air pollution impacts, sensitivity to pollution is not widely seen as a valued component of biodiversity in itself. To ensure that the metric was chosen according to a robust evidential basis, we used an innovative combination of qualitative and quantitative techniques to canvass and analyse the views of a set of key informants. To our knowledge, this is the first time that such an approach has been applied to select a biodiversity indicator. We focused on national conservation policy experts to elucidate the views of those most actively involved in policy formulation and implementation, as outlined below. The study was based in the UK, where N pollution has had severe effects on biodiversity [45,46], but the approach and results are widely relevant to studies of the impacts of environmental change on biodiversity.

## Materials and Methods

### Interviews with key informants

The interviews, including preparation and data-analysis stages, conformed to the “consolidated criteria for reporting qualitative studies” [47]. We used a purposive or ‘judgement’ sampling approach [48], identifying the 16 key individuals (13 male, 3 female) from governmental organisations in England, Scotland, Wales and Northern Ireland that have statutory responsibilities for making decisions about the strategy for managing and monitoring particular habitats. These habitat specialists had expertise in one or more of the three major habitat types that were targeted in the study. Our aim was to develop a widely-applicable metric, so we selected specialists across a range of quite different habitat types, as defined at EUNIS Level 1: D: mires, bogs and fens; E: grasslands and lands dominated by forbs, mosses or lichens; and F: heathland, scrub and tundra. These habitats are widespread and clearly affected by N pollution [46], in contrast to woodlands, for which the evidence of N impacts is less consistent [49]. The specialists were contacted initially via e-mail, which included a briefing note to provide background to the project, and invited to participate in a semi-structured interview lasting around one hour, and a subsequent ranking exercise of habitat examples to be carried out in their own time. Of 16 specialists contacted, two declined citing time pressures, but 14 were able to participate, representing the majority of the key individuals identified. Interviews were conducted between 28^th^ August and 5^th^ September 2013 by Rowe (PhD, male) and Ford (PhD, female) in person at the participants’ places of work, apart from one telephone interview. Rowe has had training in and experience of participatory methods [50], and Ford has considerable experience of qualitative research in the context of nature conservation, including conducting in-depth and semi-structured interviews, surveys, focus groups, and analyses of stakeholder attitudes, values and participation [51]. Rowe had participated in the CCE process for around 10 years prior to the study, and was fully aware of the need to define a one-dimensional HQ metric, but did not have prior assumptions about the basis for such a metric. The participants were made aware that the purpose of the research was not to focus on aspects of the habitat that are sensitive to N, but to understand how overall HQ is assessed in practice. The study did not include vulnerable participants, so we did not seek consent from an institutional review board, but we took several steps to ensure that ethical standards were upheld including: i) prior to the interviews, we provided participants with a letter explaining the purpose of the study and how it would be conducted; ii) prior to the interviews, we provided participants with a letter of consent, explaining that the data from the interviews would be confidential and how the data would be used, which the participants read and signed; iii) we stated in the letter of consent and at the start of the interview that participants could withdraw from the study at any time; iv) the data are held securely and only accessible to the researchers who undertook the interviews; and v) publications resulting from the study maintain the anonymity and confidentiality of the participants.

A set of six topics that may be related to the assessment of HQ was defined in advance, and acted as our interview guide (Table 1). Although using pre-defined topics was inevitably somewhat normative, it was thought useful to remind the specialists of a wide range of potential considerations, and to ensure some consistency of coverage. The interviews were conducted in an informal style and did not cover the topics in a prescribed sequence, to allow for variation in questioning including additional questions and two-way dialogue. This approach retained the benefits of having a clear structure to aid the analysis process and keep the research focused, whilst also providing the flexibility needed to collect rich qualitative data. Habitat specialists were asked to describe the main features that are looked for when assessing HQ (Topic 1). For Topic 2, they were asked whether some species should be valued more than others, and to explore the basis for such evaluations. Within this topic, the specialists were also asked whether invasive species (whether native or non-native) should be considered negative *per se* or only when they supplant existing species. For Topic 3, the habitat specialists were asked what plant and lichen species would lead them to rank a site as having high or low HQ. The specialists were asked whether HQ can be assessed on the basis of presence or abundance of just plants and lichens, and if not, what other taxonomic groups are important (Topic 4); and whether species-groups (e.g. forbs, subshrubs, graminoids, grasses, mosses, *Sphagna*) are useful for assessing HQ (Topic 5). The specialists were also asked their opinion on a reference community approach to assessing HQ, where a site is measured against an ideal or target example of the community (Topic 6).

**Table 1:** Pre-defined topics covered by semi-structured interviews, and themes that emerged within these topics.

Topic (pre-defined)	Theme (emergent)
T1. Main features of habitat quality	*a) Combination of features*
	*b) Habitat structure*
	*c) Vegetation composition and structure*
	*d) Geographical and temporal variability*
	*e) Ecosystem services*
	*f) Applicability and practicality*
T2. Value of individual species	*a) Structural and functional species*
	*b) Scarce species*
	*c) Invasive species*
	*d) Historical context*
	*e) Comparative values of species*
T3. Plant & lichen indicator-species	*a) Characteristics of positive indicator-species*
	*b) Characteristics of negative indicator-species*
	*c) Context of indicator-species*
T4. Taxa other than plants and lichens	*a) Importance of other taxa*
	*b) Management conflicts*
	*c) Barriers to using other taxa*
	*d) Proxy indicators of suitability for other taxa*
T5. Species-groups	*a) Pros and cons of using species-groups*
	*b) Identifying useful species-groups*
T6. Reference communities	*a) Defining a reference community*
	*b) Potential reference community definitions*

The habitat specialists’ responses were analysed under predefined topic headings (Table 1). The interviews were recorded digitally and transcribed the same day. Field notes were taken for reflection towards the end of the interview, to ensure that all topics were covered, but these notes were not used for analysis. Interview transcriptions were analysed by Ford using Atlas Ti [52]. This software facilitates marking-up of the transcripts under different topics, and subsequent collation and analysis of related text sections. Data were first coded according to each of the pre-defined six topics and by habitat (the ‘*a priori* codes’). Within each of the six topics, themes were then identified using an approach based on grounded theory, in which themes are allowed to emerge from the data, i.e. are not pre-determined by the researcher [53]. The pre-defined topics and the emergent themes are presented in Table 1. Participants were not asked to check transcriptions, but were supplied with a more extensive summary of responses than is presented here, and given a chance to respond and make corrections to interpretations and emphases.

### Ranking exercise

#### Habitat examples

Following the semi-structured interview, each habitat specialist was given a set of 12 examples for each habitat or habitats that they were responsible for. Specialists ranked a mean of 1.91 +/- 0.94 (standard deviation) sets each. The examples were taken from a database of 31,261 relevés originally used to develop the British National Vegetation Classification (NVC, [54]), and for the habitats considered in the current study were usually derived from 2×2 m relevés. All relevés were automatically assigned to the nearest NVC subcommunity using the MAVIS program [55] which uses Czekanowski’s quantitative index of similarity, i.e. taking into account the abundance as well as presence of species [56]. These NVC subcommunities were mapped onto EUNIS [57] Level 3 and Level 2 classes using established correspondences [58]. The NVC dataset was collected with the aim of sampling the full range of British habitats, including the best examples of particular habitat types, as well as from more modified sites. To ensure that this range was adequately represented by the examples, we calculated a preliminary metric for each example as the sum of its rank-scores for two simple measures: species-richness, and the inverse of the prevalence (proportion of UK 10×10 km gridsquares containing the species) of the most scarce species present. The habitat examples were ordered according to this preliminary metric, and one example was chosen at random from each of 12 strata. Each example was presented to the specialists as a list of all the plant and lichen species present in a defined area, with associated cover-score (Domin) values. Species were listed in descending order of abundance without distinguishing vascular plants, bryophytes and lichens.

#### Ranking by specialists

The specialists were asked to rank the examples in order of “overall habitat quality”, by weighing different considerations and using their overall judgement, in the same way that they would when assessing whether to give a site a conservation designation or assessing a site using the concept of “favourable habitat condition” [59]. The specialists were asked to consider this ranking carefully and return the results at a later date (all rankings were returned within two weeks of the interview). It was explained that the examples should be assessed in relation to the definition of the habitat class in question. For example, a relatively species-rich relevé with very low sub-shrub cover would not be considered a high-quality example of a heathland, since sub-shrubs define heathland. The habitat specialists were given a free choice as to whether to rank examples at EUNIS Level 2 (e.g. D1 Raised and blanket bogs) or Level 3 (e.g. D1.1 Raised bogs). In one case the specialist first classified the set into two different types and then ranked each type separately, so we assigned the top-ranked and bottom-ranked example of each type an equal overall ranking and gave the other examples an overall ranking based on their ranking within the type, i.e. assuming that both types were of equal value. A total of 21 rankings was obtained, from nine habitat-classes (Table 2).

**Table 2:** Types of habitat considered in the study, and numbers of rankings obtained.

EUNIS Level 2		*n*	EUNIS Level 3		*n*
D1	Raised and blanket bogs	3	D1.2	Blanket bogs	1
D2	Valley mires, poor fens and transition mires	1			
E1	Dry grasslands	3			
E2	Mesic grasslands	2			
E3	Seasonally wet and wet grasslands	2			
F4	Temperate shrub heathland	5	F4.1	Wet temperate shrub heathland	2
			F4.2	Dry temperate shrub heathland	2

Habitat types were defined using the EUNIS system [57].

*n* = number of specialists who ranked the set of examples for the habitat.

#### Comparisons with algorithmic metrics

The specialist’s ranking of the examples was viewed as a definitive assessment of their overall HQ. When more than one specialist ranked a set of examples, the mean rank-order was calculated. Methods used to calculate different algorithmic metrics from the same examples are summarised in Table 3, with additional information below. All metrics were designed to be positively correlated with habitat quality. Each metric was assessed according to how well they correlated with the specialist’s rank order for the same examples, using Kendall’s Tau test.

**Table 3:** Algorithmic metrics calculated from example relevé data.

Metric	Summary of calculation method
Species-richness	Total number of vascular plant, bryophyte and lichen species present.
Simpson’s diversity index	1 − (sum of squared cover proportions)
Scarcity	−1 × number of 10×10 km squares in the UK where the species occurs [60].
Positive indicator-species	Number of positive indicator-species present.
Negative indicator-species	−1 × number of negative indicator-species present.
Positive minus negative indicator-species	Number of positive indicator-species present, minus number of negative indicator-species present.
Species-groups (bog)	Total cover of *Sphagnum* species.
Species-groups (heathland)	Total cover of sub-shrubs.
Species-groups (grassland)	Forb cover / total cover.
Maximum similarity	Maximum Czekanowski similarity to a reference NVC subcommunity.
Mean similarity	Mean Czekanowski similarity to reference NVC subcommunities.
Infertility indicator-score	−1 × mean Ellenberg N score for plant species present, not cover-weighted.

All cover-based metrics were calculated by conversion of Domin scores [61] to the midpoint cover percentage of each class, i.e. Domin 1 = 1%, 2 = 2%, 3 = 3%, 4 = 7%, 5 = 18%, 6 = 29.5%, 7 = 42%, 8 = 63%, 9 = 83%, 10 = 95.5%. For each example, the value of Simpson’s Diversity Index *D*_*s*_ was calculated as

$$D_{s}=1-\sum_{1}^{i}{\left(\frac{a_{i}}{A}\right)}^{2}$$ (1)

Where *a*_*i*_ is the cover value for species *i* and *A* is the total cover for all species.

Reference communities, with high nature conservation value, were derived from the NVC [54] by considering all NVC subcommunities considered to 'overlap with', be 'equal to' or be 'contained in' the EUNIS class, using a pre-existing correspondence table [58]. Similarities of each habitat example to corresponding NVC types were calculated using the Czekanowski quantitative index [62,63], i.e.

$$CzI=100\frac{\sum_{i}^{p}2\;min(y_{ij},y_{ik})}{\sum_{i}^{p}\left(y_{ij}+y_{ik}\right)}$$ (2)

where y_ij_ and y_ik_ denote the cover of species *i* in the habitat example (*j*) and the reference community (*k*), respectively and *p* is the number of species. This index lies in the range between 0 and 1, and is 1 if the example and reference are identical. Since NVC tables include species from a large number of relevés they do not indicate the likely composition of a single relevé, so pseudo-relevés were generated for each NVC class for the similarity calculation, as explained in Tipping et al. [64]. For most EUNIS classes, several corresponding NVC types are listed, and it would be hard to justify selecting one of these as more valuable than another. We therefore applied two methods for calculating a similarity metric: the maximum similarity of the example to any corresponding NVC class; and the mean similarity to all corresponding NVC classes.

Positive and negative indicator species for each habitat (see **S1 File**) were obtained from the Common Standards Monitoring (CSM) guidance documents [65–68], which are used to assess the condition of designated sites in the UK. The guidance includes lists of indicator-species, grouped into those whose presence (and sometimes abundance or prevalence) indicates favourable condition, referred to as positive indicator-species, and species that indicate unfavourable condition, i.e. negative indicator-species. Indicator-species were originally selected on the basis that they are typical or distinctive for the habitat; are useful for determining site condition; are not so scarce that they will rarely be observed; and occur across a wide geographic range (Richard Jefferson, *pers*. *com*.). There is not an exact match between the habitat classes used in CSM and the EUNIS classes for which metrics need to be derived, so CSM guidance for the most relevant class was selected for each EUNIS Level 3 habitat. For EUNIS Level 2 habitats the lists of indicator-species for component EUNIS Level 3 habitats were combined, although some species were excluded since they appeared as both positive and negative indicators for different sub-types of the habitat in question.

The cover proportions of species-groups such as grasses, forbs or subshrubs are an important aspect of habitat structure. Different species-groups are considered important for the structure and function of different habitats. *Sphagnum* moss species have important roles in water retention and peat formation in bogs. Heathlands are defined by their subshrub component, so we calculated total cover of subshrubs, including Ericaceae, *Myrica*, *Ulex minor*, *U*. *gallii* and all *Genista*, but excluding *Ulex europaeus*, all *Rubus* and all *Salix*. Forb cover is considered a useful condition measure for grasslands [69]. The metric we calculated was forb cover / total cover, which is more mathematically robust than grass / forb cover ratio and increases with greater forb cover. Forbs were assumed to be all herbs apart from *Graminae*, and included sedges and allies (e.g. *Eriophorum* and *Luzula*), rushes, horsetails and ferns, and partially woody genera such as *Helianthemum* and *Hypericum*. Taxa not included as forbs were grasses, trees, shrubs and subshrubs (including *Rubus* and *Myrica*), mosses and lichens.

Vascular plant and bryophyte species have been scored according to where they are likely to occur in relation to different environmental axes. These scores were originally assigned by Ellenberg [70] and here we refer to them as ‘Ellenberg’ scores, but in fact we used scores derived algorithmically from UK vascular plant and bryophyte occurrence data by Mark Hill and colleagues [71,72]. Mean values of the Ellenberg ‘N’ (E_N_) score correspond to plant productivity [73,74], and in survey data have been shown to be correlated with N deposition rate [6,75].

## Results

### Qualitative analysis of interview responses

The habitat specialists’ interview responses were analysed based on six pre-defined topic headings and 22 themes (see Table 1). The key messages relating to each of these themes are summarised in Table 4, along with example supporting quotations. A more detailed summary, including further quotations, is provided in the **S2 File**.

**Table 4:** Key messages from semi-structured interviews.

Theme	Key message	Example quotations
**Topic 1: Main features of habitat quality**		
**1.a) Combination of features**	Habitat quality is viewed in terms of vegetation composition, but also more holistically as the result of a combination of features, including habitat structure and physical attributes such as water table dynamics.	*“Species composition would be the most obvious one*, *both in terms of species that are there and species that aren’t*, *relative proportions of those species… And broadly*, *the impacts of land management …”* ***I4 Heaths*, *Wetlands*, *Grasslands***
**1.b) Habitat structure**	Structural and functional aspects of habitats, such as water quality and quantity, surface topography, and management impacts, are highly important for wetlands in the assessment of habitat quality, but may also be of increasing importance in the future for other habitats.	*“…it comes back to the functionality of the habitat*. *If the habitat isn’t functioning and in three dimensional way*, *just a two dimensional approach to looking at it*, *then you will end up where you just have species disappearing*, *because you’re not taking into account the dynamism of that habitat”* ***I2 Wetlands***
**1.c) Vegetation composition and structure**	Vegetation, both in terms of composition and structure, is the dominant factor in habitat quality assessment for grasslands and heathlands. Species assemblages are typically more important for habitat quality assessment than individual species, although both can act as a proxy for environmental conditions.	*“The [vegetation] structure is one of the important things*. *…So we don’t want to see the whole site very homogeneous looking*, *mature or degenerate*, *but a diversity of the stages*.*”* ***I13 Heaths;*** *“If it's unique …that adds to your conservation value*.*”* ***I8 Grasslands;*** *“Generally [we’re] not looking for specific species*, *looking more for diversity of a certain level*.*”* ***I1 Grasslands***
**1.d) Geographical and temporal variability**	Habitat quality assessment may need to reflect geographical differences in condition–whether caused naturally or by historical anthropogenic causes–as well as the temporally dynamic changes that may occur in a habitat.	*“There would obviously be altitudinal*, *geographical*, *bio-geographical differences as well*.*”* ***I4 Heaths*, *Wetlands*, *Grasslands;*** *“It will have to quite flexible within that to take into account local variation … the way we look is not flexible enough*, *it’s too rigid*, *it’s not dynamic–habitats are dynamic*.*”* ***I2 Wetlands***
**1.e) Ecosystem services**	Ecosystem services, such as water and climate regulation, have the potential be included as an additional factor to biodiversity conservation objectives in habitat quality assessments.	*“If you’re faced with choices…I would prefer that that total resource had the capacity to deliver a number of key services*, *of which biodiversity is not necessarily the most important*. *If I’m looking at individual sites then the biodiversity is important in that it is part of the value of that site to society*. *But I wouldn’t expect all bog or peatlands to have that*.*”* ***I3 Wetlands***
**1.f) Applicability and practicality**	The Common Standards Monitoring guidance acts as the key framework for much of the habitat quality assessment; however, tailoring of CSM indicator-species lists has improved local applicability and practicality for use by local monitoring officers.	“*When the JNCC Common Standards were published we wrote our own …translation of it*, *just added a bit more flesh to the bones really*, *and perhaps made it a little bit less generic*.*”* ***I6 Wetlands***
**Topic 2: Value of individual species**		
**2.a) Structural and functional species**	Species that are structurally or functionally important have particular value, especially in wetland habitats. They may have increasing relevance to other habitats in the face of climate change.	*“We see* Sphagnum *as a priority for the accumulation of peat*, *basically*.*”* ***I11b Wetlands;*** *“So a priority for us is that with climate warming we’re trying to get bogs to function naturally so they are then more resilient to warming”* ***I11b Wetlands***
**2.b) Scarce species**	Scarce species provide added value to a habitat, and can be important for site designation. However, they are not usually a dominant criterion for assessing habitat quality, in part because they do not occur on enough sites to be widely applicable as indicators.	*“… we tried to avoid things which were not particularly common or quite rare*, *because although they might be telling you that where they occur that that’s an absolutely perfect site*, *because the hydrology of the soils or whatever is right*, *they are not very useful in terms of an overall assessment of the condition of a site*.*”* ***I10 Grasslands***
**2.c) Invasive species**	Invasive species, whether native or non-native, are generally considered negative when they out-compete or cause other detrimental impacts to valued native species, rather than being considered negative *per se*. Feasibility of removal, and whether invasion is a natural part of range expansion, are also taken into consideration.	“*…what is wrong about alien species? The thing that’s wrong about them is that they can become invasive and take over from native vegetation*. *So if they are doing that then that’s bad*, *but if they are not*, *they’re just there at very low cover*, *then from a vegetation point of view I don’t think you’d worry*.*”* ***I8 Grasslands***
**2.d) Historical context**	The historical context of a habitat or a particular site can influence the management goals with regards to species assemblage, potentially resulting in over-valuing or undervaluing species.	*“I can think of heathlands in this area*, *lowland heathland*, *where we now have very scarce species*, *but they could be historically quite widespread…*. *Things like* Viola lactea *… those kind of species*, *which are associated with a certain set of structures within the heathland*. *So scarce species can be important because they are actually typical*.*”* ***I5 Heaths***
**2.e) Comparative values of species**	Valuing some species more highly than others has challenges and potential conflicts, for example over which species to conserve.	*“The public view of grasslands is not necessarily our view of grasslands*.*”* ***I1 Grasslands***
**Topic 3: Plant & lichen indicator-species**		
**3.a) Characteristics of positive indicator-species**	Criteria for selecting positive plant and lichen indicators include being distinctive for the habitat, typical for the habitat, or indicating good environmental conditions.	*“I suppose we are looking for those particular species which are niche species of that particular habitat”* ***I2 Wetlands;*** *“I mean*, *basically we tried to select those species that are really indicative in telling you the conditions are right for the maintenance of that grassland…”* ***I10 Grasslands***
**3.b) Characteristics of negative indicator-species**	Negative indicator-species are typically those that out-compete desirable native species, but they also may be those that indicate poor environmental conditions such as heavy grazing and eutrophication. Some species may become negative indicators if they cause ecosystem disbenefits.	*“The worst negative indicators are the ones that take up most space*. *And then species that react to high nutrient levels*.* *.* *. *So it’s species that take up space at the expense of a greater variety of non-competitive things*.*”* ***I1 Grasslands;*** *“*Eriophorum vaginatum *is one of these species that transports methane to the atmosphere*. *So the fact that we know that it’s shunting all this methane up into the atmosphere at the moment is maybe not quite so good*.*”****I3 Wetlands***
**3.c) Context of indicator-species**	The use of species-indicators can be complex and requires flexibility to take into account variation in geographical factors (including scale and altitude), natural habitat variation, and other factors such as past management.	*“I think the subshrub depends on where you are*, *what your soils are*, *and to a certain extent*, *past management*.*”* ***I5 Heaths***
**Topic 4: Taxa other than plants and lichens**		
**4.a) Importance of other taxa**	Plants and lichens are typically considered more useful for the assessment of habitat quality than other taxa. However, other taxa can be an important feature for site designation, in which case the species will typically be monitored by specialists in those taxa rather than as part of routine habitat quality assessment.	*“If it’s an SSSI and it’s designated for the habitat and also the birds or invertebrates*, *then somebody would look at the population trends or there will be some monitoring of other species*, *but I*, *or the training I give to the advisors*, *doesn’t include directly the invertebrates or birds*. *But they are very important*.*”* ***I13 Heaths***
**4.b) Management conflicts**	In some cases other taxa require management conditions that are not compatible with high habitat quality; however these different requirements can normally be accommodated, particularly on larger sites.	*“Golden plover and blanket bog is probably the classic example … the issue would be some of the sites where golden plover is a feature*, *as well as the blanket bog*, *and to manage the blanket bog for the golden plover would effectively render it unfavourable as far as blanket bog condition is concerned*.*”* ***I9 Wetlands***
**4.c) Barriers to using other taxa**	There are a number of barriers to using other taxa in habitat quality assessment, including limitations in resources, time, skill, knowledge of species’ autecology, and consistency of sightings.	*“… you are dependent on the weather conditions when you go out*, *it’s very much on what we see*, *so I think all these species they are important but it would be very difficult to record them on a consistent basis”*. ***I1 Grasslands***
**4.d) Proxy indicators for suitability of other taxa**	The quality of a habitat with respect to other taxa may be inferred through using environmental conditions, such as habitat structure and vegetation composition, as a proxy.	“*Our role is as habitat specialists*. *And we look at structure*, *so we look at the height of vegetation*, *and we look at the ages of ericoids*, *and we look at bare ground*, *so you look at elements of the habitat that invertebrates or reptiles might find useful or interesting*. *But our colleagues would be expected to pick that up*.*”* ***I11a Heaths*, *Wetlands*, *Grasslands***
**Topic 5: Species-groups**		
**5.a) Pros and cons of using species-groups**	Assessing cover of species-groups can be a useful tool for inferring habitat quality. However, species-groups may not always provide the level of detail necessary, for example for rare subcommunities or as a proxy for environmental conditions.	*“…it’s actually quite a useful check that you’ve made your original estimation quite good”* ***I9 Wetlands*, *Heaths;*** *“…we would definitely be thinking about the amount of* Arctostaphylos *that there is in those examples of the habitat*, *rather than just covering dwarf shrubs”* ***I4 Heaths***
**5.b) Identifying useful species-groups**	Cover of species-groups can be useful in habitat quality assessment, such as forbs and grasses for grasslands; dwarf shrubs, graminoids, mosses and lichens for heathlands; and mosses for wetlands, but a group such as ‘graminoids’ can include negative and positive indicator-species.	*“You could go just in terms of groups if you don’t want a full list*, *which will change a lot from site to site*, *so just looking at ericoids*, *graminoids*, *forbs and yeah non vascular species like mosses*, *that grouping could be useful*.*”* **I13 Heaths**
**Topic 6: Reference communities**		
**6.a) Defining a reference community**	There is considerable variation in the examples of each habitat that are seen as high quality, so it would be very difficult to define a reference community.	*“…as soon as you start thinking about a reference community*, *you start thinking*, *well*, *there are all these exceptions*.*”* ***I4 Heaths*, *Wetlands;*** *“*.* *.* *.*we want a broader view than that*, *so I don’t quite like NVC held up as an example of what a grassland should be*.*”* ***I1 Grasslands***
**6.b) Potential reference community definitions**	The NVC tables, or past records where these exist, could be used to define a reference community at site level, or a set of reference communities covering the variation in high-quality habitat.	“*I think the NVC is probably the closest you’re going to get to have something that we all agree on that is relatively close to that single reference point*, *but around it there needs to be that grey area of a little bit of flexibility as well*.* *.* *.*”* ***I2 Wetlands;*** *“If you actually had old records for the site and could go back and compare*, *that would be very useful…but impractical*.*”* ***I1 Grasslands***

The interview data highlight the complexity surrounding specialists’ assessment of habitat quality. Various features of habitats have importance in assessing quality, including habitat functioning and ecosystem services delivery, vegetation composition and structure, cover of species-groups, and presence or abundance of individual species. Habitat type, geographical context (e.g. scale, altitude and location) and historical context and management may all also affect judgements of quality. As expected, some species are valued more highly than others, such as those with a significant structural or functional role for the habitat, or scarce species–although scarce species have a limited role in overall habitat quality assessment, principally because they are found on few sites. Species considered to be indicators of low habitat quality are typically those that are invasive (native or non-native) and have detrimental impacts on valued species; or species that directly indicate poor environmental conditions such as inappropriate management. Conversely, the presence of species that indicate good environmental conditions and/or are distinctive for the habitat is seen as indicating high habitat quality. Plants and lichen species are typically considered more useful than other taxa for assessing habitat quality; although other taxa can be useful, there are practical constraints in using them widely. Our analysis also revealed that varied and distinct examples may be considered of high quality within a particular habitat type, for example because of local variation, so it would be challenging to identify a suitable reference community for a given habitat.

### Comparison of specialists’ and algorithmic rankings

**Fig 1:**
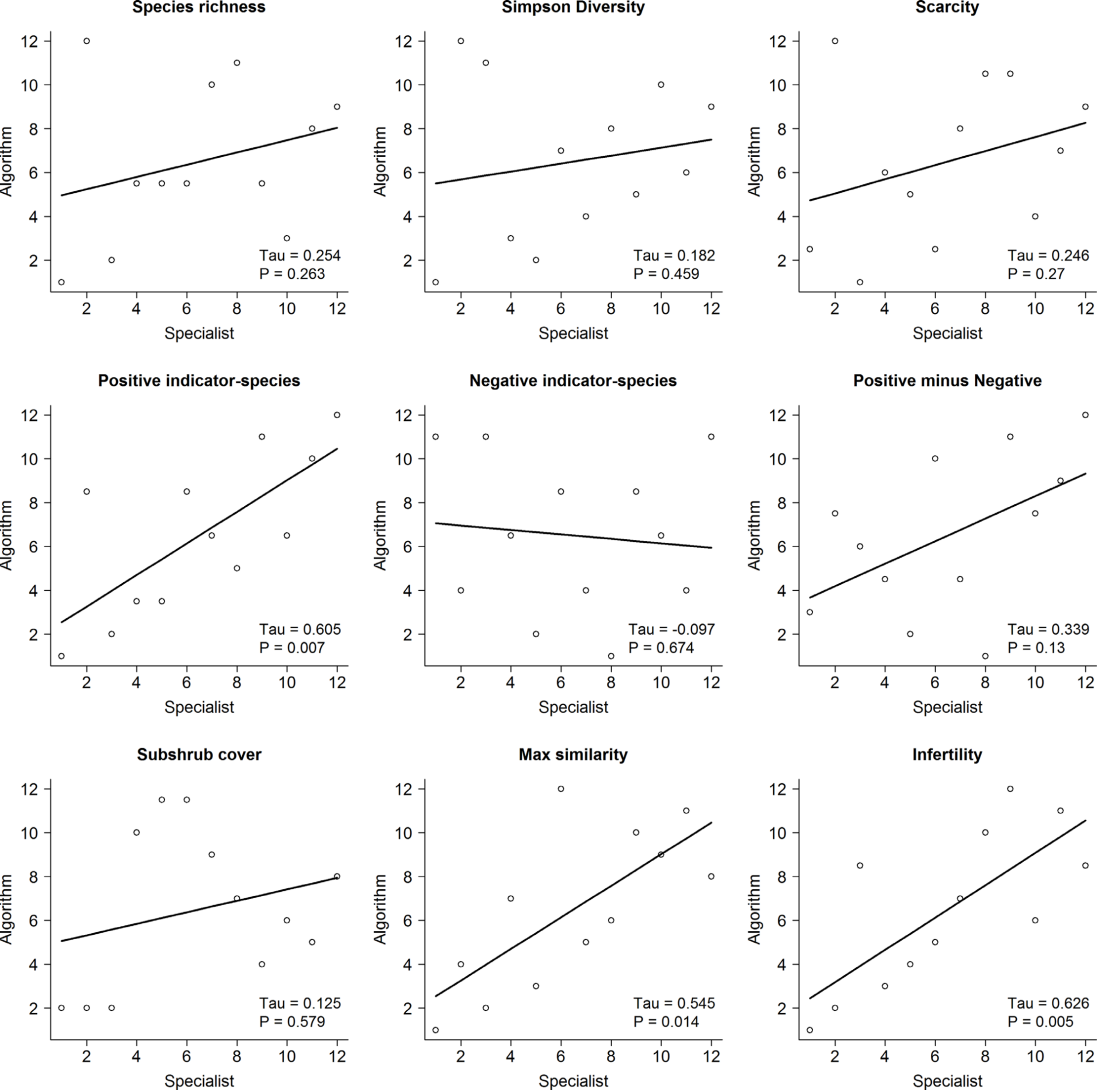
Correlations of habitat specialists’ rankings with algorithmic rankings, for heathland. Correlations between rank-scores given by habitat specialists to 12 examples of EUNIS class F4 (Temperate shrub heathland) and rank scores for metrics based on algorithms applied to the same examples: Species richness; Simpson diversity; Scarcity, −1 × UK prevalence of least-prevalent species present; number of positive indicator-species; −1 × number of negative indicator-species; number of positive indicator-species minus number of negative indicator-species; subshrub cover; greatest Czekanowski similarity to corresponding National Vegetation Classification (NVC) subcommunities; Infertility, −1 × mean Ellenberg N score. Tau, Kendall’s Tau statistic.

The correlations between the mean ranks assigned by specialists and the ranks of the same set of examples according to different metrics are illustrated for the habitat for which the most rankings were obtained (F4, Temperate shrub heathland) in Fig 1, and summarised for all habitats in Table 5. For example, assessing examples of heathland using the number of positive indicator-species resulted in a closer correlation with specialists’ rankings than did ranking by species-richness (Fig 1).

**Table 5:** Coefficients for correlations between habitat specialists’ rankings of examples of different habitats and algorithmic rankings.

Metric	D1	D1.2	D2	E1	E2	E3	F4	F4.1	F4.2
	Correlation coefficient (Kendall’s Tau)								
SR	0.29^ns^	0.21^ns^	0.06^ns^	0.52*	0.50*	0.81***	0.25^ns^	0.80***	0.60**
SimpsonD	0.39^ns^	0.02^ns^	0.08^ns^	0.47*	0.48*	0.46*	0.18^ns^	0.54*	0.17*
Scarcity	0.38^ns^	0.08^ns^	0.03^ns^	0.27^ns^	0.02^ns^	0.37^ns^	0.25^ns^	0.06^ns^	0.02^ns^
Positive	0.85***	0.09^ns^	−0.40^ns^	0.72***	0.81***	0.85***	0.61**	0.78***	0.52*
Negative	0.13^ns^	−0.25^ns^	−0.13^ns^	−0.18^ns^	0.32^ns^	−0.12^ns^	−0.10^ns^	−0.35^ns^	−0.36^ns^
Pos—Neg	0.84***	0.04^ns^	−0.46*	0.74***	0.66**	0.74***	0.34^ns^	0.67**	0.55*
Subshrub							0.12^ns^	0.29^ns^	0.39^ns^
Forb/Tot				0.02^ns^	0.39^ns^	0.15^ns^			
Sphagnum	0.53*	0.62*	0.52*						
MaxSimil	0.58**	0.43^ns^	0.29^ns^	0.63**	0.48*	0.12^ns^	0.54*	0.64**	0.08^ns^
MeanSimil	0.42^ns^	0.71**	0.29^ns^	0.53*	0.61**	0.58**	0.30^ns^	0.63**	0.30^ns^
Infertility	0.49*	0.25^ns^	0.11^ns^	0.47*	0.73***	0.36^ns^	0.63**	0.57*	−0.05^ns^

SR, Species-richness; SimpsonD, Simpson’s Diversity Index; Scarcity, (−1 × UK prevalence of least-prevalent species present); Positive, number of positive indicator-species; Negative, −1 × number of negative indicator-species; Pos − Neg, number of positive indicator-species minus number of negative indicator-species; MaxSimil, greatest Czekanowski similarity to corresponding National Vegetation Classification (NVC) subcommunities; MeanSimil, mean Czekanowski similarity to corresponding NVC subcommunities; Infertility, −1 × mean Ellenberg N score; ns, not significant

*, *P* < 0.05

**, *P* < 0.01

***, *P* < 0.001; blank cells, not applicable.

The number of positive indicator-species was the metric most consistently associated with specialists’ rankings with correlations in seven of the nine classes, and was the most closely correlated metric in six of the nine cases. Subtracting the number of negative indicator-species mainly did not improve the correlation, with the exception of D2 (valley mires, poor fens and transition mires). Species-richness was correlated with specialists’ rankings in grassland habitats and was the most closely correlated of all metrics for the two heathland subclasses, although there was no correlation for the overall heathlands class. Simpson’s diversity index was correlated for five of the nine habitat classes, although only at marginal significance levels. Values for the scarcity metric were not correlated. Both maximum and mean similarity to corresponding NVC communities were significantly correlated with specialists’ rankings for five classes, and mean similarity was the most closely correlated of all metrics for D1.2 (blanket bog).

The cover of functional groups was only significantly correlated with specialists’ rankings in the case of *Sphagnum* in the bog and mire classes. The cover of subshrubs in heathlands was not correlated with heathland HQ. The lack of correlation with cover ratio of forbs in grasslands can be explained by examples with large cover values for species typical of fertile and/or disturbed swards.

Similarity to reference assemblages was significantly correlated with HQ for most sets of grassland examples and for dry heathland, although not for bogs. In some cases the correlation was closer when using similarity to the most-similar reference assemblage, but in some cases the mean similarity to all reference assemblages for the habitat class better reflected HQ. The Infertility index was strongly correlated with HQ in E2 ‘Mesic grasslands’, and was significantly correlated in four of the other habitat classes, but the association was not as consistent and clear as it was for positive indicator-species.

## Discussion

Priorities as to what aspects of biodiversity should be protected are inevitably subjective, despite considerable efforts to evaluate biodiversity metrics objectively (e.g. [76,77]). Drawing upon social research methods to explore the opinions of conservation professionals allows a more meaningful and justified analysis than can be obtained using only natural science approaches [78]. The use of semi-structured interviews necessitated a small sample size, but was appropriate for the research objectives as it provided an insight into the value judgements of key individuals [48], in this case those responsible for designating, managing and monitoring habitats. Although our sample was not extensive enough to ensure data saturation, due to logistical constraints, it nevertheless captured views of a range of key specialists with decision-making roles. It proved very useful to discuss biodiversity concepts and issues before the ranking exercise, and in particular to explain that the study aimed to explore the basis for assessing HQ in general rather than assessing sensitivity to N in particular. The overall pattern of correlations with alternative metrics (Table 5) corroborated the qualitative analysis, and provided additional information which allows metrics to be assessed. Rank-correlation has been used previously to assess different biodiversity metrics in relation to the first axis of a plant community ordination [79], but to our knowledge this is the first study to apply qualitative and quantitative methods to assess the relationship between different biodiversity metrics and the priorities of conservation professionals.

The interview responses revealed that vegetation composition is very important for assessing habitat condition. Taxa other than plants and lichens are clearly important, but practical constraints mean that vegetation composition is the most commonly used basis for discriminating between sites (Table 4). This implies that presenting data on floristic composition did not misrepresent the examples. It is advantageous when assessing biotic integrity to have data for different taxon groups [80], but where such data are lacking it is necessary to consider surrogate taxa. Plants are important for habitat structure and energy inputs, and provide a diversity of chemical substrates and toxins likely to influence biodiversity at other trophic levels. Effects of environmental drivers on other taxa are often mediated by vegetation–for example, declines in butterfly abundance driven by N pollution are likely due to the loss of suitable micro-sites in more rank vegetation [81]. Plants are also suitable surrogate taxa for assessing overall biodiversity since they are easily observed and expertise in their identification is comparatively widespread [82], as also noted by the respondents.

The procedure for assigning examples to habitats was related to the algorithmic metrics based on Czekanowski similarity, with the difference that the assignment was binary (in or out of the habitat class) whereas the metric was continuous. Also, a combination of species-richness and species scarcity was used for the preliminary stratification, to ensure a broad range of examples. Other metrics were not directly related to the methods used to select examples. It seems unlikely that the methods used to assign and stratify the examples could have led to circularity in choosing one of the related metrics, even if that had been the outcome of the study. Species scarcity was not significantly correlated with the specialists’ assessments for any habitat, and species richness was the most strongly correlated for only two of the nine habitat classes. The method for presenting the examples was limited, in that a visual impression was not presented. Not all of the topics and themes that emerged in discussions were represented by the examples–in particular biophysical aspects, the size of the habitat stand, and characteristics of the site rather than the individual habitat. However, the main aim of the study was to assess metrics for within-habitat biodiversity, for which species lists with abundances provide adequate information. Photographs would have provided additional insight into the habitat, but were not available for the example relevés, and in any case are difficult to standardise since the appearance of a stand depends on the species in flower, the light conditions, and the skills of the photographer. Another consideration was that ultimate aim of the study was to allow interpretation of model outputs, and the current generation of pollution impacts models is unable to generate visual impressions of different plant assemblages.

Species richness is clearly related to public perceptions of biodiversity value, although its limitations as a biodiversity metric are recognised in the scientific literature [83]. Species richness may not reflect phylogenetic richness, nor the presence of rare species [84]. An increase in species richness may reflect the invasion of atypical species [9], and this concern emerged frequently in the interviews, particularly when discussing bogs and heaths (see **S2 File)**.

Scarce species were an important priority for the specialists, but were considered impractical to use for site assessment. Reflecting this, the national prevalence of the most-scarce plant species present was not related to overall assessments. Schemes for assessing sites by combining species-weights based on scarcity (e.g. [85]) have proved difficult to implement, principally due to disagreements over the weighting that should be given to different species [86]. Metrics based on similarity to a reference assemblage were also generally rejected by the specialists, mostly on the basis that there was too much variation in what is considered an ideal or target species composition for a particular habitat class. Rankings based on similarity to a reference were well-correlated with specialists’ rankings for some of the habitats, but this correlation not consistent, e.g. there was no correlation for F4.2 ‘Dry temperate shrub heathland’. Mean fertility score (Ellenberg N) has been shown to be correlated with N deposition rate [45], and rankings based on mean Ellenberg N were correlated with specialists’ rankings for five of the nine habitat classes, but was the most highly correlated of the metrics only for one class, F4 ‘Temperate shrub heathland’.

The cover of functionally important groups was highlighted as an important factor by many of the specialists. However, rankings based on the total cover of these functionally important groups did not correspond to the specialists’ rankings, with the exception of *Sphagnum* cover for bogs. *Sphagnum* is important for the maintenance of bog function, and *Sphagnum* cover was correlated with HQ, albeit weakly, for all three of the bog / mire classes assessed. Functionally important groups did not appear to be important for assessment of the other two classes studied, even though total forb cover is frequently cited as indicating better-quality grasslands, and subshrub cover is definitive for heathland. Functional-group abundance may be important for specific habitats, but this metric was only superior to that based on positive indicator-species in two cases, and may not be as widely applicable.

The specialists referred frequently and favourably to positive indicator-species, and a metric based on the number of positive indicator-species gave the most consistent correlation with the specialists’ rankings. Methods used to select indicator species are often insufficiently clear [87,88], but indicator-species are commonly used in ecological assessment (e.g. [89]) and the current study suggests that this use is appropriate. The presence of a few key taxa has been shown to be a good predictor of total species richness across many taxa [90]. The positive indicator-species used in the study were originally selected on the basis that they are typical of and distinctive for the habitat, and indicate favourable site conditions [91]. These aspects help explain why correlations with overall habitat quality were relatively strong for this metric. In particular, the aspect of distinctiveness–species that are not necessarily scarce, but are restricted to good-quality examples of the habitat–emerged as critical. Similarly, in a study of Brazilian sites, Trindade et al. [92] found that the best indicators of mammal diversity were species with restricted ranges.

Negative indicator-species were also discussed in many of the interviews, although they were commonly seen as indicating poor site conditions rather than being damaging in their own right. Although some negative indicator species indicate that the abiotic environment is changing adversely for the habitat, non-native invasive species in particular can invade sites even when environmental conditions are unchanged. The distinct responses of these two groups may explain the lack of correlation between the number of negative indicator-species in the examples and the specialists’ HQ rankings.

The study demonstrated that despite the diverse criteria used to assess the quality of a range of habitats across the varied conditions of the UK, it is possible to find a single metric that gives an indication of other aspects of habitat quality. The results from the study were instrumental in the adoption by a recent CCE Workshop of habitat suitability for characteristic species as a common indicator for use in air pollution impacts modelling by all Signatory Parties [93]. The specific metric selected in this study is likely to be applicable to only some ecosystems, policy contexts, and environmental threats. However, the approach taken proved capable of providing sufficient evidence to support a course of action in a complex and much-debated policy area. The combination of qualitative and quantitative methods used to summarise expert judgements is likely to be effective at other interfaces between science and policy.

## Conclusions

The evidence provided by analysis of the habitat specialists’ responses suggests that the presence of positive indicator-species is the most suitable basis for a biodiversity metric for use in the context of evaluating habitat damage and recovery from air pollution. The metric most consistently related to overall habitat quality was the number of positive indicator-species present. Models are available which predict the habitat-suitabilities for a large number of plant and lichen species under different pollution scenarios [13], and predicting suitability for positive indicator-species is a useful focus for this modelling work. The success of positive-indicator species in indicating overall quality suggests that identifying species that discriminate good-quality examples of a habitat should be a priority for conservation assessment. The combined quantitative and qualitative approach taken to determining an appropriate metric is likely to be widely applicable in other fields where it is necessary to summarise expert judgements.

## Supporting Information

S1 File Indicator species. (DOCX)

S2 File Extensive summary of interviews. (DOCX)
