# Supplementary material for: Using Qualitative and Quantitative Methods to Choose a Habitat Quality Metric for Air Pollution Policy Evaluation
Source: PLoS One. 2016 Aug 24;11(8):e0161085. doi: 10.1371/journal.pone.0161085 (PMC4996518; doi:10.1371/journal.pone.0161085)
Supplement: S1 File — (DOCX) [file pone.0161085.s001.docx]

**S1 Indicator species**

The following species were collated from text descriptions and indicator-species lists in the summary tables contained in Common Standards Monitoring Guidance documents issued by the UK Joint Nature Conservation Committee (see http://www.jncc.gov.uk/page-2201). Positive indicator-species are designated ‘P’ and negative indicator-species are designated ‘N’. The names and species codes shown are those used by the UK Biological Records Centre. Habitats were classified according to the EUNIS system (see <http://eunis.eea.europa.eu/habitats>), i.e.:

| D1 | Raised and blanket bogs |
| --- | --- |
| D2 | Valley mires, poor fens and transition mires |
| E1 | Dry grasslands |
| E2 | Mesic grasslands |
| E3 | Seasonally wet and wet grasslands |
| F4 | Temperate shrub heathland |
| D1.1 | Raised bogs |
| D1.2 | Blanket bogs |
| E1.2 | Perennial calcareous grassland and basic steppes |
| E1.7 | Closed non-Mediterranean dry acid and neutral grassland |
| E2.1 | Permanent mesotrophic pastures and aftermath-grazed meadows |
| E2.2 | Low and medium altitude hay meadows |
| E3.4 | Moist or wet eutrophic and mesotrophic grassland |
| E3.5 | Moist or wet oligotrophic grassland |
| F4.1 | Wet heaths |
| F4.2 | Dry heaths |

| BRC_name | BRC_number | D1 | D2 | E1 | E2 | E3 | F4 | D1.1 | D1.2 | E1.2 | E1.7 | E2.1 | E2.2 | E3.4 | E3.5 | F4.1 | F4.2 |
| --- | --- | --- | --- | --- | --- | --- | --- | --- | --- | --- | --- | --- | --- | --- | --- | --- | --- |
| *Acer pseudoplatanus (c)* | 9205 |  |  | N |  | N |  |  |  | N |  |  |  | N | N |  |  |
| *Aceras anthropophorum* | 9206 |  |  | P | P | P |  |  |  | P | P | P | P | P | P |  |  |
| *Achillea ptarmica* | 9209 |  |  |  |  | P |  |  |  |  |  |  |  | P | P |  |  |
| *Acinos arvensis* | 92012 |  |  | P |  |  |  |  |  | P |  |  |  |  |  |  |  |
| *Agrimonia eupatoria* | 92022 |  |  | P | P |  |  |  |  | P |  | P | P |  |  |  |  |
| *Agrostis canina* | 92035 |  |  |  |  |  | N |  |  |  |  |  |  |  |  |  | N |
| *Agrostis canina sens.lat.* | 92035 |  |  |  |  |  | N |  |  |  |  |  |  |  |  |  | N |
| *Agrostis capillaris* | 92040 | N |  |  |  |  | N | N | N |  |  |  |  |  |  | N | N |
| *Agrostis curtisii* | 92038 |  |  | P |  |  | N |  |  |  | P |  |  |  |  |  | N |
| *Agrostis gigantea* | 92036 |  |  |  |  |  | N |  |  |  |  |  |  |  |  |  | N |
| *Agrostis stolonifera* | 92039 |  |  |  |  |  | N |  |  |  |  |  |  |  |  |  | N |
| *Agrostis vinealis* | 92035.1 |  |  |  |  |  | N |  |  |  |  |  |  |  |  |  | N |
| *Aira caryophyllea* | 92041 |  |  | P |  |  |  |  |  | P | P |  |  |  |  |  |  |
| *Aira praecox* | 92042 |  |  | P |  |  | P |  |  | P | P |  |  |  |  |  | P |
| *Alchemilla alpina* | 92048 |  |  | P | P |  |  |  |  | P |  | P | P |  |  |  |  |
| *Alchemilla glabra* | 92051 |  |  | P | P |  |  |  |  | P |  | P | P |  |  |  |  |
| *Alchemilla vulgaris agg.* | 92058 |  |  | P | P |  |  |  |  | P |  | P | P |  |  |  |  |
| *Alnus glutinosa (c)* | 92077 |  |  |  |  |  | N |  |  |  |  |  |  |  |  | N |  |
| *Ammophila arenaria* | 92097 |  |  |  |  |  | N |  |  |  |  |  |  |  |  |  | N |
| *Anacamptis pyramidalis* | 92098 |  |  | P | P | P |  |  |  | P | P | P | P | P | P |  |  |
| *Anagallis tenella* | 920100 |  |  |  |  | P | P |  |  |  |  |  |  | P | P | P |  |
| *Andromeda polifolia* | 920103 | P |  |  |  |  | P | P | P |  |  |  |  |  |  | P |  |
| *Anenome nemorosa* | 920105 |  |  | P | P |  |  |  |  |  | P | P | P |  |  |  |  |
| *Angelica sylvestris* | 920109 |  |  | P |  | P |  |  |  | P |  |  |  | P | P |  |  |
| *Antennaria dioica* | 920116 |  |  | P |  |  |  |  |  | P |  |  |  |  |  |  |  |
| *Anthriscus sylvestris* | 920125 |  |  | N |  | N |  |  |  | N |  |  |  | N | N |  |  |
| *Anthyllis vulneraria* | 920126 |  |  | P |  |  |  |  |  | P |  |  |  |  |  |  |  |
| *Aphanes arvensis* | 920131 |  |  | P |  |  |  |  |  | P | P |  |  |  |  |  |  |
| *Apium nodiflorum* | 920137 |  |  |  |  |  | N |  |  |  |  |  |  |  |  | N |  |
| *Arbutus unedo* | 920149 |  |  | N |  |  |  |  |  | N |  |  |  |  |  |  |  |
| *Arctostaphylos alpinus* | 920156 | P |  |  |  |  | P | P | P |  |  |  |  |  |  | P | P |
| *Arctostaphylos uva-ursi* | 920155 | P |  |  |  |  | P | P | P |  |  |  |  |  |  | P | P |
| *Armeria maritima* | 920166 |  |  | P |  |  | P |  |  | P |  |  |  |  |  |  | P |
| *Arrhenatherum elatius* | 920169 |  |  | N | N | N |  |  |  | N | N | N | N | N | N |  |  |
| *Asperula cynanchica* | 9205472 |  |  | P |  |  |  |  |  | P |  |  |  |  |  |  |  |
| *Astragalus danicus* | 920207 |  |  | P |  |  |  |  |  | P | P |  |  |  |  |  |  |
| *Aulacomnium palustre* | 82042 |  | P |  |  |  |  |  |  |  |  |  |  |  |  |  |  |
| *Bellis perennis* | 920231 |  |  | N |  |  |  |  |  | N | N |  |  |  |  |  |  |
| *Berberis vulgaris* | 920232 |  |  | N |  |  |  |  |  | N |  |  |  |  |  |  |  |
| *Berula erecta* | 920234 |  |  |  | P |  |  |  |  |  |  | P | P |  |  |  |  |
| *Betula nana* | 920238 | P |  |  |  |  | P | P | P |  |  |  |  |  |  | P | P |
| *Betula pendula* | 920239 | N |  |  |  |  | N | N | N |  |  |  |  |  |  | N | N |
| *Betula pubescens* | 920240 | N |  |  |  |  | N | N | N |  |  |  |  |  |  | N | N |
| *Betula spp.* | 9204445 | N |  |  |  |  | N | N | N |  |  |  |  |  |  | N | N |
| *Brachypodium pinnatum* | 920249 |  |  | N |  |  |  |  |  | N |  |  |  |  |  |  |  |
| *Briza media* | 920256 |  |  | P |  |  |  |  |  | P |  |  |  |  |  |  |  |
| *Bromus erectus* | 920263 |  |  | N |  |  |  |  |  | N |  |  |  |  |  |  |  |
| *Bromus hordeaceus* | 920269 |  |  |  | N |  |  |  |  |  |  | N | N |  |  |  |  |
| *Calluna vulgaris* | 920309 | P |  | P |  | P | P | P | P |  | P |  |  | P | P | P | P |
| *Caltha palustris* | 920310 |  |  |  | P | P |  |  |  |  |  | P | P | P | P |  |  |
| *Campanula glomerata* | 920315 |  |  | P |  |  |  |  |  | P |  |  |  |  |  |  |  |
| *Campanula rotundifolia* | 920322 |  |  | P |  |  |  |  |  | P | P |  |  |  |  |  |  |
| *Campylopus subulatus* | 820147 |  |  | N |  |  |  |  |  | N |  |  |  |  |  |  |  |
| *Carduus acanthoides* | 920335 |  |  | N |  |  |  |  |  | N |  |  |  |  |  |  |  |
| *Carduus nutans* | 920337 |  |  | N |  |  |  |  |  | N | N |  |  |  |  |  |  |
| *Carex atrata* | 920345 |  |  | P |  |  | N |  |  | P |  |  |  |  |  | P | N |
| *Carex atrofusca* | 920346 |  |  | P |  |  | N |  |  | P |  |  |  |  |  | P | N |
| *Carex bigelowii* | 920349 | P |  | P |  |  | N | P | P | P |  |  |  |  |  | P | N |
| *Carex binervis* | 920350 |  |  | P |  |  | N |  |  | P |  |  |  |  |  | P | N |
| *Carex buxbaumii* | 920352 |  |  | P |  |  | N |  |  | P |  |  |  |  |  | P | N |
| *Carex capillaris* | 920353 |  |  | P |  |  | N |  |  | P |  |  |  |  |  | P | N |
| *Carex caryophyllea* | 920355 |  |  | P |  |  | N |  |  | P |  |  |  |  |  | P | N |
| *Carex chordorrhiza* | 920356 |  |  | P |  |  | N |  |  | P |  |  |  |  |  | P | N |
| *Carex curta* | 920359 |  |  | P |  |  | N |  |  | P |  |  |  |  |  | P | N |
| *Carex depauperata* | 920362 |  |  | P |  |  | N |  |  | P |  |  |  |  |  | P | N |
| *Carex digitata* | 920364 |  |  | P |  |  | N |  |  | P |  |  |  |  |  | P | N |
| *Carex dioica* | 920365 |  |  | P |  |  | N |  |  | P |  |  |  |  |  | P | N |
| *Carex distans* | 920366 |  |  | P |  |  | N |  |  | P |  |  |  |  |  | P | N |
| *Carex disticha* | 920367 |  |  | P |  |  | N |  |  | P |  |  |  |  |  | P | N |
| *Carex divisa* | 920368 |  |  | P |  |  | N |  |  | P |  |  |  |  |  | P | N |
| *Carex echinata* | 920370 |  | P | P |  |  | N |  |  | P |  |  |  |  |  | P | N |
| *Carex ericetorum* | 920373 |  |  | P |  |  | N |  |  | P |  |  |  |  |  | P | N |
| *Carex filiformis* | 920375 |  |  | P |  |  | N |  |  | P |  |  |  |  |  | P | N |
| *Carex flacca* | 920376 |  |  | P |  |  | N |  |  | P |  |  |  |  |  | P | N |
| *Carex hostiana* | 920382 |  |  | P |  |  | N |  |  | P |  |  |  |  |  | P | N |
| *Carex humilis* | 920383 |  |  | P |  |  | N |  |  | P |  |  |  |  |  | P | N |
| *Carex lachenalii* | 920384 |  |  | P |  |  | N |  |  | P |  |  |  |  |  | P | N |
| *Carex lasiocarpa* | 920386 |  | P |  |  |  |  |  |  |  |  |  |  |  |  |  |  |
| *Carex limosa* | 920388 |  |  | P |  |  | N |  |  | P |  |  |  |  |  | P | N |
| *Carex magellanica* | 920403 |  |  | P |  |  | N |  |  | P |  |  |  |  |  | P | N |
| *Carex microglochin* | 920390 |  |  | P |  |  | N |  |  | P |  |  |  |  |  | P | N |
| *Carex nigra* | 920393 |  | P | P |  |  | N |  |  | P |  |  |  |  |  | P | N |
| *Carex ornithopoda* | 920395 |  |  | P |  |  | N |  |  | P |  |  |  |  |  | P | N |
| *Carex ovalis* | 920397 |  |  | P |  |  | N |  |  | P |  |  |  |  |  | P | N |
| *Carex panicea* | 920400 |  | P | P |  |  | N |  |  | P |  |  |  |  |  |  | N |
| *Carex pauciflora* | 920402 |  |  | P |  |  | N |  |  | P |  |  |  |  |  | P | N |
| *Carex pilulifera* | 920405 |  |  | P |  |  | N |  |  | P |  |  |  |  |  | P | N |
| *Carex pulicaris* | 920408 |  |  | P |  |  | N |  |  | P |  |  |  |  |  |  | N |
| *Carex punctata* | 920409 |  |  | P |  |  | N |  |  | P |  |  |  |  |  | P | N |
| *Carex rariflora* | 920410 |  |  | P |  |  | N |  |  | P |  |  |  |  |  | P | N |
| *Carex recta* | 920411 |  |  | P |  |  | N |  |  | P |  |  |  |  |  | P | N |
| *Carex rostrata* | 920414 |  | P |  |  |  |  |  |  |  |  |  |  |  |  |  |  |
| *Carex rupestris* | 920415 |  |  | P |  |  | N |  |  | P |  |  |  |  |  | P | N |
| *Carex saxatilis* | 920417 |  |  | P |  |  | N |  |  | P |  |  |  |  |  | P | N |
| *Carex spicata* | 920357 |  |  | P |  |  | N |  |  | P |  |  |  |  |  | P | N |
| *Carex strigosa* | 920420 |  |  | P |  |  | N |  |  | P |  |  |  |  |  | P | N |
| *Carex sylvatica* | 920421 |  |  | P |  |  | N |  |  | P |  |  |  |  |  | P | N |
| *Carex trinervis* | 920422 |  |  | P |  |  | N |  |  | P |  |  |  |  |  | P | N |
| *Carex vaginata* | 920423 |  |  | P |  |  | N |  |  | P |  |  |  |  |  | P | N |
| *Carex vesicaria* | 920424 |  |  | P |  |  | N |  |  | P |  |  |  |  |  | P | N |
| *Carex viridula subsp.brachyrrhyncha* | 920387 |  |  | P |  |  | N |  |  | P |  |  |  |  |  | P | N |
| *Carex viridula subsp.oedocarpa* | 920361 |  | P | P |  |  | N |  |  | P |  |  |  |  |  | P | N |
| *Carex viridula subsp.viridula* | 9207118 |  |  | P |  |  | N |  |  | P |  |  |  |  |  | P | N |
| *Carex vulpinoidea* | 920426 |  |  | P |  |  | N |  |  | P |  |  |  |  |  | P | N |
| *Carlina vulgaris* | 920427 |  |  | P |  |  |  |  |  | P |  |  |  |  |  |  |  |
| *Carum verticillatum* | 920431 |  |  |  |  | P |  |  |  |  |  |  |  | P | P |  |  |
| *Centaurea nigra* | 920444 |  |  | P | P | P |  |  |  | P |  | P | P | P | P |  |  |
| *Centaurea scabiosa* | 920446 |  |  | P |  |  |  |  |  | P |  |  |  |  |  |  |  |
| *Centaurium erythraea* | 9205486 |  |  | P |  |  |  |  |  | P | P |  |  |  |  |  |  |
| *Centranthus ruber* | 920455 |  |  | N |  |  |  |  |  | N |  |  |  |  |  |  |  |
| *Cephalanthera damasonium* | 920457 |  |  | P | P | P |  |  |  | P | P | P | P | P | P |  |  |
| *Cephalanthera longifolia* | 920458 |  |  | P | P | P |  |  |  | P | P | P | P | P | P |  |  |
| *Cephalanthera rubra* | 920459 |  |  | P | P | P |  |  |  | P | P | P | P | P | P |  |  |
| *Cerastium fontanum* | 920467 |  |  | N |  |  |  |  |  | P | N |  |  |  |  |  |  |
| *Cetraria chlorophylla* | 5502842 |  |  | P |  |  |  |  |  |  | P |  |  |  |  |  |  |
| *Cetraria commixta* | 5502843 |  |  | P |  |  |  |  |  |  | P |  |  |  |  |  |  |
| *Cetraria cucullata* | 5502844 |  |  | P |  |  |  |  |  |  | P |  |  |  |  |  |  |
| *Cetraria glauca* | 5505299 |  |  | P |  |  |  |  |  | P | P |  |  |  |  |  |  |
| *Cetraria hepatizon* | 5502847 |  |  | P |  |  |  |  |  | P | P |  |  |  |  |  |  |
| *Cetraria islandica* | 5502848 |  |  | P |  |  |  |  |  | P | P |  |  |  |  |  |  |
| *Cetraria nivalis* | 5502851 |  |  | P |  |  |  |  |  | P | P |  |  |  |  |  |  |
| *Cetraria pinastri* | 5502852 |  |  | P |  |  |  |  |  | P | P |  |  |  |  |  |  |
| *Cetraria sepincola* | 5502853 |  |  | P |  |  |  |  |  | P | P |  |  |  |  |  |  |
| *Chamanerion angustifolium* | 920477 |  |  | N |  |  | N |  |  | N | N |  |  |  |  |  | N |
| *Cirsium acaule* | 920514 |  |  | P |  |  |  |  |  | P |  |  |  |  |  |  |  |
| *Cirsium arvense* | 920515 | N |  | N | N | N | N | N | N | N | N | N | N | N | N | N | N |
| *Cirsium dissectum* | 920516 |  |  |  | P | P |  |  |  |  |  | P | P | P | P |  |  |
| *Cirsium heterophyllum* | 920518 |  |  | P | P |  |  |  |  | P |  | P | P |  |  |  |  |
| *Cirsium palustre* | 920520 |  | P |  |  |  |  |  |  |  |  |  |  |  |  |  |  |
| *Cirsium vulgare* | 920522 | N |  | N | N | N | N | N | N | N | N | N | N | N | N |  | N |
| *Cladonia acuminata* | 5502858 | P |  | P |  |  | P | P | P | P | P |  |  |  |  | P |  |
| *Cladonia alcicornis* | 5505226 | P |  | P |  |  | P | P | P | P | P |  |  |  |  | P |  |
| *Cladonia alpestris* | 5505300 | P |  | P |  |  | P | P | P | P | P |  |  |  |  | P |  |
| *Cladonia alpicola* | 5505324 | P |  | P |  |  | P | P | P | P | P |  |  |  |  | P |  |
| *Cladonia amaurocraea* | 5502859 | P |  | P |  |  | P | P | P | P | P |  |  |  |  | P |  |
| *Cladonia arbuscula* | 5502860 | P |  | P |  |  | P | P | P | P | P |  |  |  |  | P |  |
| *Cladonia bacillaris* | 5502861 | P |  | P |  |  | P | P | P | P | P |  |  |  |  | P |  |
| *Cladonia bellidiflora* | 5502862 | P |  | P |  |  | P | P | P | P | P |  |  |  |  | P |  |
| *Cladonia caespiticia* | 5502864 | P |  | P |  |  | P | P | P | P | P |  |  |  |  | P |  |
| *Cladonia cariosa* | 5502865 | P |  | P |  |  | P | P | P | P | P |  |  |  |  | P |  |
| *Cladonia carneola* | 5502866 | P |  | P |  |  | P | P | P | P | P |  |  |  |  | P |  |
| *Cladonia cenotea* | 5502867 | P |  | P |  |  | P | P | P | P | P |  |  |  |  | P |  |
| *Cladonia cervicornis* | 5502868 | P |  | P |  |  | P | P | P | P | P |  |  |  |  | P |  |
| *Cladonia cf.coccifera* | 5505338 | P |  | P |  |  | P | P | P | P | P |  |  |  |  | P |  |
| *Cladonia cf.polydactyla* | 5505246 | P |  | P |  |  | P | P | P | P | P |  |  |  |  | P |  |
| *Cladonia cf.subcervicornis* | 5505339 | P |  | P |  |  | P | P | P | P | P |  |  |  |  | P |  |
| *Cladonia chlorophaea agg.* | 5505327 | P |  | P |  |  | P | P | P | P | P |  |  |  |  | P |  |
| *Cladonia ciliata* | 5502871 | P |  | P |  |  | P | P | P | P | P |  |  |  |  | P |  |
| *Cladonia coccifera* | 5502873 | P |  | P |  |  | P | P | P | P | P |  |  |  |  | P |  |
| *Cladonia coniocraea* | 5502874 | P |  | P |  |  | P | P | P | P | P |  |  |  |  | P |  |
| *Cladonia conista* | 5505301 | P |  | P |  |  | P | P | P | P | P |  |  |  |  | P |  |
| *Cladonia convoluta* | 5502875 | P |  | P |  |  | P | P | P | P | P |  |  |  |  | P |  |
| *Cladonia cornuta* | 5502876 | P |  | P |  |  | P | P | P | P | P |  |  |  |  | P |  |
| *Cladonia crispata* | 5502877 | P |  | P |  |  | P | P | P | P | P |  |  |  |  | P |  |
| *Cladonia cyathomorpha* | 5502880 | P |  | P |  |  | P | P | P | P | P |  |  |  |  | P |  |
| *Cladonia deformis* | 5502881 | P |  | P |  |  | P | P | P | P | P |  |  |  |  | P |  |
| *Cladonia degenerans* | 5505302 | P |  | P |  |  | P | P | P | P | P |  |  |  |  | P |  |
| *Cladonia delessertii* | 5505349 | P |  | P |  |  | P | P | P | P | P |  |  |  |  | P |  |
| *Cladonia destricta* | 5505334 | P |  | P |  |  | P | P | P | P | P |  |  |  |  | P |  |
| *Cladonia digitata* | 5502882 | P |  | P |  |  | P | P | P | P | P |  |  |  |  | P |  |
| *Cladonia ecmocyna* | 5505340 | P |  | P |  |  | P | P | P | P | P |  |  |  |  | P |  |
| *Cladonia fimbriata* | 5502883 | P |  | P |  |  | P | P | P | P | P |  |  |  |  | P |  |
| *Cladonia floerkeana* | 5502885 | P |  | P |  |  | P | P | P | P | P |  |  |  |  | P |  |
| *Cladonia foliacea* | 5502886 | P |  | P |  |  | P | P | P | P | P |  |  |  |  | P |  |
| *Cladonia furcata* | 5502888 | P |  | P |  |  | P | P | P | P | P |  |  |  |  | P |  |
| *Cladonia glauca* | 5502890 | P |  | P |  |  | P | P | P | P | P |  |  |  |  | P |  |
| *Cladonia gonecha* | 5505303 | P |  | P |  |  | P | P | P | P | P |  |  |  |  | P |  |
| *Cladonia gracilis* | 5502891 | P |  | P |  |  | P | P | P | P | P |  |  |  |  | P |  |
| *Cladonia impexa* | 5505304 | P |  | P |  |  | P | P | P | P | P |  |  |  |  | P |  |
| *Cladonia incrassata* | 5502894 | P |  | P |  |  | P | P | P | P | P |  |  |  |  | P |  |
| *Cladonia leucophaea* | 5505305 | P |  | P |  |  | P | P | P | P | P |  |  |  |  | P |  |
| *Cladonia luteoalba* | 5502895 | P |  | P |  |  | P | P | P | P | P |  |  |  |  | P |  |
| *Cladonia macilenta* | 5502896 | P |  | P |  |  | P | P | P | P | P |  |  |  |  | P |  |
| *Cladonia macrophylla* | 5502897 | P |  | P |  |  | P | P | P | P | P |  |  |  |  | P |  |
| *Cladonia mitis* | 5502902 | P |  | P |  |  | P | P | P | P | P |  |  |  |  | P |  |
| *Cladonia nylanderi* | 5505306 | P |  | P |  |  | P | P | P | P | P |  |  |  |  | P |  |
| *Cladonia ochrochlora* | 5502903 | P |  | P |  |  | P | P | P | P | P |  |  |  |  | P |  |
| *Cladonia papillaria* | 5505307 | P |  | P |  |  | P | P | P | P | P |  |  |  |  | P |  |
| *Cladonia parasitica* | 5502904 | P |  | P |  |  | P | P | P | P | P |  |  |  |  | P |  |
| *Cladonia pityrea* | 5505308 | P |  | P |  |  | P | P | P | P | P |  |  |  |  | P |  |
| *Cladonia pocillum* | 5502908 | P |  | P |  |  | P | P | P | P | P |  |  |  |  | P |  |
| *Cladonia polydactyla* | 5502909 | P |  | P |  |  | P | P | P | P | P |  |  |  |  | P |  |
| *Cladonia portentosa* | 5502910 | P |  | P |  |  | P | P | P | P | P |  |  |  |  | P |  |
| *Cladonia pyxidata* | 5502912 | P |  | P |  |  | P | P | P | P | P |  |  |  |  | P |  |
| *Cladonia ramulosa* | 5502913 | P |  | P |  |  | P | P | P | P | P |  |  |  |  | P |  |
| *Cladonia rangiferina* | 5502914 | P |  | P |  |  | P | P | P | P | P |  |  |  |  | P |  |
| *Cladonia rangiformis* | 5502915 | P |  | P |  |  | P | P | P | P | P |  |  |  |  | P |  |
| *Cladonia scabriuscula* | 5505309 | P |  | P |  |  | P | P | P | P | P |  |  |  |  | P |  |
| *Cladonia squamosa* | 5502919 | P |  | P |  |  | P | P | P | P | P |  |  |  |  | P |  |
| *Cladonia strepsilis* | 5502923 | P |  | P |  |  | P | P | P | P | P |  |  |  |  | P |  |
| *Cladonia subcervicornis* | 5502925 | P |  | P |  |  | P | P | P | P | P |  |  |  |  | P |  |
| *Cladonia subrangiformis* | 5505285 | P |  | P |  |  | P | P | P | P | P |  |  |  |  | P |  |
| *Cladonia subsquamosa* | 5505286 | P |  | P |  |  | P | P | P | P | P |  |  |  |  | P |  |
| *Cladonia subulata* | 5502926 | P |  | P |  |  | P | P | P | P | P |  |  |  |  | P |  |
| *Cladonia tenuis* | 5505322 | P |  | P |  |  | P | P | P | P | P |  |  |  |  | P |  |
| *Cladonia turgida* | 5502929 | P |  | P |  |  | P | P | P | P | P |  |  |  |  | P |  |
| *Cladonia uncialis* | 5502931 | P |  | P |  |  | P | P | P | P | P |  |  |  |  | P |  |
| *Cladonia verticillata* | 5505287 | P |  | P |  |  | P | P | P | P | P |  |  |  |  | P |  |
| *Clinopodium vulgare* | 920530 |  |  | P |  |  |  |  |  | P |  |  |  |  |  |  |  |
| *Cochlearia pyrenaica* | 9205422 |  |  | P |  |  |  |  |  | P |  |  |  |  |  |  |  |
| *Coeloglossum viride* | 920537 |  |  | P | P | P |  |  |  | P | P | P | P | P | P |  |  |
| *Conopodium majus* | 920541 |  |  |  | P |  |  |  |  |  |  | P | P |  |  |  |  |
| *Corallorhiza trifida* | 920545 |  |  | P | P | P |  |  |  | P | P | P | P | P | P |  |  |
| *Cornus suecica* | 920478 | P |  |  |  |  |  | P | P |  |  |  |  |  |  |  |  |
| *Corynephorus canescens* | 920558 |  |  |  |  |  | P |  |  |  |  |  |  |  |  |  | P |
| *Cotoneaster spp.* | 9204526 |  |  | N |  |  |  |  |  | N |  |  |  |  |  |  |  |
| *Crataegus monogyna (s)* | 920569 |  |  | N |  | N |  |  |  | N |  |  |  | N | N |  |  |
| *Crepis paludosa* | 920576 |  |  |  |  | P |  |  |  |  |  |  |  | P | P |  |  |
| *Cynosurus cristatus* | 920597 |  |  | N |  |  |  |  |  | N | N |  |  |  |  |  |  |
| *Cypripedium calceolus* | 920601 |  |  | P | P | P |  |  |  | P | P | P | P | P | P |  |  |
| *Cytisus scoparius* | 9201822 |  |  |  |  |  | N |  |  |  |  |  |  |  |  |  | N |
| *Dactylis glomerata* | 920607 |  |  | N | N | N |  |  |  | N | N | N | N | N | N |  |  |
| *Dactylorhiza fuchsii* | 920608 |  |  | P | P | P |  |  |  | P | P | P | P | P | P |  |  |
| *Dactylorhiza incarnata* | 920609 |  |  | P | P | P |  |  |  | P | P | P | P | P | P |  |  |
| *Dactylorhiza lapponica* | 9202964 |  |  | P | P | P |  |  |  | P | P | P | P | P | P |  |  |
| *Dactylorhiza maculata* | 920610 |  |  | P | P | P |  |  |  | P | P | P | P | P | P |  |  |
| *Dactylorhiza majalis* | 920611 |  |  | P | P | P |  |  |  | P | P | P | P | P | P |  |  |
| *Dactylorhiza praetermissa* | 920612 |  |  | P | P | P |  |  |  | P | P | P | P | P | P |  |  |
| *Dactylorhiza purpurella* | 920613 |  |  | P | P | P |  |  |  | P | P | P | P | P | P |  |  |
| *Dactylorhiza spp.* | 9204528 |  |  |  | P |  |  |  |  |  |  | P | P |  |  |  |  |
| *Dactylorhiza traunsteineri* | 920614 |  |  | P | P | P |  |  |  | P | P | P | P | P | P |  |  |
| *Danthonia decumbens* | 9201915 |  |  | P |  |  | N |  |  | P |  |  |  |  |  |  | N |
| *Deschampsia cespitosa* | 920627 | N |  |  | N | N |  | N | N |  |  | N | N | N | N |  |  |
| *Deschampsia flexuosa* | 920628 |  |  | N |  |  | N |  |  |  | N |  |  |  |  |  | N |
| *Dianthus deltoides* | 920635 |  |  | P |  |  |  |  |  | P |  |  |  |  |  |  |  |
| *Digitalis purpurea* | 920640 |  |  |  |  |  | N |  |  |  |  |  |  |  |  | N | N |
| *Draba incana* | 920651 |  |  | P |  |  |  |  |  | P |  |  |  |  |  |  |  |
| *Drosera intermedia* | 920655 | P |  |  |  |  | P | P | P |  |  |  |  |  |  | P |  |
| *Drosera longifolia* | 920654 | P |  |  |  |  | P | P | P |  |  |  |  |  |  | P |  |
| *Drosera rotundifolia* | 920657 | P |  |  |  |  | P | P | P |  |  |  |  |  |  | P |  |
| *Dryas octopetala* | 920658 |  |  | P |  |  |  |  |  | P |  |  |  |  |  |  |  |
| *Eleocharis acicularis* | 920673 |  |  |  |  |  | N |  |  |  |  |  |  |  |  | N |  |
| *Eleocharis austriaca* | 9202267 |  |  |  |  |  | N |  |  |  |  |  |  |  |  | N |  |
| *Eleocharis multicaulis* | 920674 |  |  |  |  |  | N |  |  |  |  |  |  |  |  | N |  |
| *Eleocharis palustris* | 920675 |  |  |  |  |  | N |  |  |  |  |  |  |  |  | N |  |
| *Eleocharis quinqueflora* | 920677 |  |  |  |  |  | N |  |  |  |  |  |  |  |  | N |  |
| *Eleocharis uniglumis* | 920678 |  |  |  |  |  | N |  |  |  |  |  |  |  |  | N |  |
| *Empetrum nigrum* | 920684 | P |  |  |  |  | P | P | P |  |  |  |  |  |  | P | P |
| *Epilobium alsinifolium* | 920690 |  |  |  |  |  | N |  |  |  |  |  |  |  |  | N | N |
| *Epilobium anagallidifolium* | 920691 |  |  |  |  |  | N |  |  |  |  |  |  |  |  | N | N |
| *Epilobium brunnescens* | 920699 |  |  |  |  |  | N |  |  |  |  |  |  |  |  | N | N |
| *Epilobium ciliatum* | 920688 |  |  |  |  |  | N |  |  |  |  |  |  |  |  | N | N |
| *Epilobium hirsutum* | 920692 | N | N |  |  |  | N | N | N |  |  |  |  |  |  | N | N |
| *Epilobium lanceolatum* | 920694 |  |  |  |  |  | N |  |  |  |  |  |  |  |  | N | N |
| *Epilobium montanum* | 920695 |  |  |  |  |  | N |  |  |  |  |  |  |  |  | N | N |
| *Epilobium obscurum* | 920696 |  |  |  |  |  | N |  |  |  |  |  |  |  |  | N | N |
| *Epilobium palustre* | 920697 |  | P |  |  |  |  |  |  |  |  |  |  |  |  |  |  |
| *Epilobium parviflorum* | 920698 |  |  |  |  |  | N |  |  |  |  |  |  |  |  | N | N |
| *Epilobium roseum* | 920700 |  |  |  |  |  | N |  |  |  |  |  |  |  |  | N | N |
| *Epilobium tetragonum* | 9207292 |  |  |  |  |  | N |  |  |  |  |  |  |  |  | N | N |
| *Epipactis atrorubens* | 920702 |  |  | P | P | P |  |  |  | P | P | P | P | P | P |  |  |
| *Epipactis helleborine* | 920705 |  |  | P | P | P |  |  |  | P | P | P | P | P | P |  |  |
| *Epipactis leptochila* | 9205476 |  |  | P | P | P |  |  |  | P | P | P | P | P | P |  |  |
| *Epipactis palustris* | 920708 |  |  | P | P | P |  |  |  | P | P | P | P | P | P |  |  |
| *Epipactis phyllanthes* | 920709 |  |  | P | P | P |  |  |  | P | P | P | P | P | P |  |  |
| *Epipactis purpurata* | 920710 |  |  | P | P | P |  |  |  | P | P | P | P | P | P |  |  |
| *Epipactis youngiana* | 9202549 |  |  | P | P | P |  |  |  | P | P | P | P | P | P |  |  |
| *Epipogium aphyllum* | 920711 |  |  | P | P | P |  |  |  | P | P | P | P | P | P |  |  |
| *Equisetum arvense* | 910712 |  |  |  | N |  |  |  |  |  |  | N | N |  |  |  |  |
| *Erica cilliaris* | 920725 | P |  |  |  |  | P | P | P |  |  |  |  |  |  | P | P |
| *Erica cinerea* | 920726 | P |  | P |  |  | P | P | P |  | P |  |  |  |  | P | P |
| *Erica tetralix* | 920731 | P |  | P |  | P | P | P | P |  | P |  |  | P | P | P | P |
| *Erica vagans* | 920732 | P |  |  |  |  | P | P | P |  |  |  |  |  |  | P | P |
| *Erigeron acer* | 920733 |  |  | P |  |  |  |  |  | P |  |  |  |  |  |  |  |
| *Eriophorum angustifolium* | 920740 | P | P |  |  |  | N | P | P |  |  |  |  |  |  | N |  |
| *Eriophorum vaginatum* | 920744 | P |  |  |  |  |  | P | P |  |  |  |  |  |  |  |  |
| *Erodium cicutarium* | 920745 |  |  | P |  |  | P |  |  | P | P |  |  |  |  |  | P |
| *Eupatorium cannabinum* | 920763 |  |  |  | P | P |  |  |  |  |  | P | P | P | P |  |  |
| *Euphrasia officinalis agg.* | 9202243 |  |  | P | P |  |  |  |  | P |  | P | P |  |  |  |  |
| *Fallopia japonica* | 9201528 |  |  |  |  |  | N |  |  |  |  |  |  |  |  | N | N |
| *Festuca altissima* | 920812 |  |  |  |  |  | N |  |  |  |  |  |  |  |  |  | N |
| *Festuca arenaria* | 920819 |  |  |  |  |  | N |  |  |  |  |  |  |  |  |  | N |
| *Festuca armoricana* | 9207359 |  |  |  |  |  | N |  |  |  |  |  |  |  |  |  | N |
| *Festuca arundinacea* | 920813 |  |  |  |  |  | N |  |  |  |  |  |  |  |  |  | N |
| *Festuca filiformis* | 920822.2 |  |  |  |  |  | N |  |  |  |  |  |  |  |  |  | N |
| *Festuca gigantea* | 920816 |  |  |  |  |  | N |  |  |  |  |  |  |  |  |  | N |
| *Festuca huonii* | 9207361 |  |  |  |  |  | N |  |  |  |  |  |  |  |  |  | N |
| *Festuca lemanii* | 9205430 |  |  |  |  |  | N |  |  |  |  |  |  |  |  |  | N |
| *Festuca longifolia* | 920817 |  |  |  |  |  | N |  |  |  |  |  |  |  |  |  | N |
| *Festuca ovina* | 920822 |  |  |  |  |  | N |  |  |  |  |  |  |  |  |  | N |
| *Festuca ovina agg.* | 920821 |  |  |  |  |  | N |  |  |  |  |  |  |  |  |  | N |
| *Festuca pratensis* | 920823 |  |  |  |  |  | N |  |  |  |  |  |  |  |  |  | N |
| *Festuca pratensis x Lolium perenne (x Festulolium loliaceum)* | 920815 |  |  |  |  |  | N |  |  |  |  |  |  |  |  |  | N |
| *Festuca rubra* | 920825 |  |  |  |  |  | N |  |  |  |  |  |  |  |  |  | N |
| *Festuca rubra agg.* | 920824 |  |  |  |  |  | N |  |  |  |  |  |  |  |  |  | N |
| *Festuca vivipara* | 920826 |  |  |  |  |  | N |  |  |  |  |  |  |  |  |  | N |
| *Filipendula ulmaria* | 920833 |  |  | P | P | P |  |  |  | P |  | P | P | P | P |  |  |
| *Filipendula vulgaris* | 920834 |  |  | P | P |  | P |  |  | P |  | P | P |  |  |  | P |
| *Fragaria vesca* | 920838 |  |  | P |  |  |  |  |  | P |  |  |  |  |  |  |  |
| *Fraxinus excelsior (c)* | 920841 |  |  | N |  |  |  |  |  | N |  |  |  |  |  |  |  |
| *Galium aparine* | 920873 |  |  | N | N |  |  |  |  | N |  | N | N |  |  |  |  |
| *Galium palustre* | 920882 |  | P |  | P | P |  |  |  |  |  | P | P | P | P |  |  |
| *Galium saxatile* | 920878 |  | P | P |  |  | P |  |  | P | P |  |  |  |  | P | P |
| *Galium sterneri* | 920883.2 |  |  | P |  |  |  |  |  | P |  |  |  |  |  |  |  |
| *Galium uliginosum* | 920887 |  |  |  | P | P |  |  |  |  |  | P | P | P | P |  |  |
| *Galium verum* | 920888 |  |  | P | P |  | P |  |  | P | P | P | P |  |  |  | P |
| *Gaultheria shallon* | 920890 |  |  |  |  |  | N |  |  |  |  |  |  |  |  |  | N |
| *Genista anglica* | 920891 |  |  |  |  |  | P |  |  |  |  |  |  |  |  | P | P |
| *Genista pilosa* | 920892 |  |  |  |  |  | P |  |  |  |  |  |  |  |  |  | P |
| *Genista tinctoria* | 920893 |  |  | P | P |  |  |  |  |  | P | P | P |  |  |  |  |
| *Gentiana verna* | 920896 |  |  | P |  |  |  |  |  | P |  |  |  |  |  |  |  |
| *Gentianella amarella* | 920897 |  |  | P |  |  |  |  |  | P |  |  |  |  |  |  |  |
| *Gentianella anglica* | 920899 |  |  | P |  |  |  |  |  | P |  |  |  |  |  |  |  |
| *Gentianella campestris* | 920901 |  |  | P |  |  |  |  |  | P |  |  |  |  |  |  |  |
| *Gentianella ciliata* | 9202629 |  |  | P |  |  |  |  |  | P |  |  |  |  |  |  |  |
| *Gentianella germanica* | 920903 |  |  | P |  |  |  |  |  | P |  |  |  |  |  |  |  |
| *Gentianella uliginosa* | 920905 |  |  | P |  |  |  |  |  | P |  |  |  |  |  |  |  |
| *Geranium sanguineum* | 920920 |  |  | P |  |  |  |  |  | P |  |  |  |  |  |  |  |
| *Geranium sylvaticum* | 920921 |  |  | P | P |  |  |  |  | P |  | P | P |  |  |  |  |
| *Geum rivale* | 920924 |  |  | P | P | P |  |  |  | P |  | P | P | P | P |  |  |
| *Glyceria fluitans* | 920933 |  |  |  |  | N | N |  |  |  |  |  |  | N | N | N |  |
| *Glyceria maxima* | 920934 | N | N |  | N | N |  | N | N |  |  | N | N | N | N |  |  |
| *Goodyera repens* | 920943 |  |  | P | P | P |  |  |  | P | P | P | P | P | P |  |  |
| *Gymnadenia conopsea* | 920948 |  |  | P | P | P |  |  |  | P | P | P | P | P | P |  |  |
| *Hammarbya paludosa* | 920951 |  |  | P | P | P |  |  |  | P | P | P | P | P | P |  |  |
| *Helianthemum appeninum* | 920593 |  |  | P |  |  |  |  |  | P |  |  |  |  |  |  |  |
| *Helianthemum canum* | 920954 |  |  | P |  |  |  |  |  | P |  |  |  |  |  |  |  |
| *Helianthemum nummularium* | 920955 |  |  | P |  |  | P |  |  | P |  |  |  |  |  |  | P |
| *Helictotrichon pubescens* | 920962 |  |  | N |  |  |  |  |  | N |  |  |  |  |  |  |  |
| *Herminium monorchis* | 920969 |  |  | P | P | P |  |  |  | P | P | P | P | P | P |  |  |
| *Himantoglossum hircinum* | 920978 |  |  | P | P | P |  |  |  | P | P | P | P | P | P |  |  |
| *Hippocrepis comosa* | 920979 |  |  | P |  |  |  |  |  | P |  |  |  |  |  |  |  |
| *Hippophaea rhamnoides* | 920980 |  |  |  |  |  | N |  |  |  |  |  |  |  |  |  | N |
| *Holcus lanatus* | 920983 | N |  | N | N | N | N | N | N | N | N | N | N | N | N | N |  |
| *Hydrocotyle vulgaris* | 920999 |  |  |  | P | P |  |  |  |  |  | P | P | P | P |  |  |
| *Hypericum hirsutum* | 9201010 |  |  | P |  |  |  |  |  | P |  |  |  |  |  |  |  |
| *Hypericum humifusum* | 9201011 |  |  | P |  |  |  |  |  | P |  |  |  |  |  |  |  |
| *Hypericum maculatum* | 9201006 |  |  | P |  |  |  |  |  | P |  |  |  |  |  |  |  |
| *Hypericum montanum* | 9201013 |  |  | P |  |  |  |  |  | P |  |  |  |  |  |  |  |
| *Hypericum perforatum* | 9201014 |  |  | P |  |  |  |  |  | P |  |  |  |  |  |  |  |
| *Hypericum pulchrum* | 9201015 |  |  | P |  |  |  |  |  | P |  |  |  |  |  |  |  |
| *Hypocheoris radicata* | 9201020 |  |  |  |  |  | P |  |  |  |  |  |  |  |  |  | P |
| *Juncus acutiflorus* | 9201050 |  | N |  | N |  | N |  |  |  |  | N | N |  |  | N |  |
| *Juncus articulatus* | 9201054 |  | N |  | N |  | N |  |  |  |  | N | N |  |  | N |  |
| *Juncus effusus* | 9201067 | N | N | N | N |  | N | N | N |  | N | N | N |  |  | N | N |
| *Juncus squarrosus* | 9201075 |  |  | N | N |  | N |  |  |  | N | N | N |  |  | N | N |
| *Knautia arvensis* | 9201084 |  |  | P |  |  |  |  |  | P |  |  |  |  |  |  |  |
| *Kobresia simpliciuscula* | 9201085 |  |  | P |  |  |  |  |  | P |  |  |  |  |  |  |  |
| *Koeleria macrantha* | 9201087 |  |  | P |  |  |  |  |  | P |  |  |  |  |  |  |  |
| *Lathyrus linifolius* | 9201112 |  |  | P | P |  |  |  |  | P | P | P | P |  |  |  |  |
| *Lathyrus pratensis* | 9201116 |  |  |  | P |  |  |  |  |  |  | P | P |  |  |  |  |
| *Leontodon hispidus* | 9201130 |  |  | P | P | P |  |  |  | P |  | P | P | P | P |  |  |
| *Leontodon saxatilis* | 9201131 |  |  | P | P |  |  |  |  | P |  | P | P |  |  |  |  |
| *Leontodon taraxacoides* | 9201131 |  |  | P |  |  |  |  |  |  | P |  |  |  |  |  |  |
| *Linum catharticum* | 9201169 |  |  | P |  |  |  |  |  | P |  |  |  |  |  |  |  |
| *Liparis loeselii* | 9201171 |  |  | P | P | P |  |  |  | P | P | P | P | P | P |  |  |
| *Listera cordata* | 9201172 |  |  | P | P | P |  |  |  | P | P | P | P | P | P |  |  |
| *Listera ovata* | 9201173 |  |  | P | P | P |  |  |  | P | P | P | P | P | P |  |  |
| *Logfia minima* | 920831 |  |  |  |  |  | P |  |  |  |  |  |  |  |  |  | P |
| *Lolium perenne* | 9201183 |  |  | N | N | N |  |  |  | N | N | N | N | N | N |  |  |
| *Lotus corniculatus* | 9201191 |  |  | P | P |  | P |  |  | P | P | P | P |  |  |  | P |
| *Lotus uliginosus* | 9201194 |  |  |  | P | P |  |  |  |  |  | P | P | P | P |  |  |
| *Luzula spicata* | 9201208 |  |  | P |  |  |  |  |  | P |  |  |  |  |  |  |  |
| *Lychnis flos-cuculi* | 9201210 |  | P |  | P | P |  |  |  |  |  | P | P | P | P |  |  |
| *Lythrum salicaria* | 9201227 |  |  |  |  | P |  |  |  |  |  |  |  | P | P |  |  |
| *Mentha aquatica* | 9201272 |  |  |  | P | P |  |  |  |  |  | P | P | P | P |  |  |
| *Menyanthes trifoliata* | 9201289 | P | P |  |  |  |  | P | P |  |  |  |  |  |  |  |  |
| *Molinia caerulea* | 9201307 |  |  |  |  |  | N |  |  |  |  |  |  |  |  | N | N |
| *Myosotis alpestris* | 9201316 |  |  | P |  |  |  |  |  | P |  |  |  |  |  |  |  |
| *Myrica gale* | 9201328 | P |  |  |  |  | P | P | P |  |  |  |  |  |  | P | P |
| *Nardus stricta* | 9201344 |  |  |  |  |  | N |  |  |  |  |  |  |  |  |  | N |
| *Narthecium ossifragum* | 9201345 | P |  |  |  | P | P | P | P |  |  |  |  | P | P | P |  |
| *Neotinea maculata* | 9201351 |  |  | P | P | P |  |  |  | P | P | P | P | P | P |  |  |
| *Neottia nidus-avis* | 9201352 |  |  | P | P | P |  |  |  | P | P | P | P | P | P |  |  |
| *Oenanthe crocata* | 9201363 |  |  |  |  |  | N |  |  |  |  |  |  |  |  | N |  |
| *Oenanthe silaifolia* | 9201368 |  |  |  | P |  |  |  |  |  |  | P | P |  |  |  |  |
| *Ophrys apifera* | 9201382 |  |  | P | P | P |  |  |  | P | P | P | P | P | P |  |  |
| *Ophrys fuciflora* | 9201383 |  |  | P | P | P |  |  |  | P | P | P | P | P | P |  |  |
| *Ophrys insectifera* | 9201384 |  |  | P | P | P |  |  |  | P | P | P | P | P | P |  |  |
| *Ophrys sphegodes* | 9201385 |  |  | P | P | P |  |  |  | P | P | P | P | P | P |  |  |
| *Orchis laxiflora* | 9201386 |  |  | P | P | P |  |  |  | P | P | P | P | P | P |  |  |
| *Orchis mascula* | 9201387 |  |  | P | P | P |  |  |  | P | P | P | P | P | P |  |  |
| *Orchis militaris* | 9201388 |  |  | P | P | P |  |  |  | P | P | P | P | P | P |  |  |
| *Orchis morio* | 9201389 |  |  | P | P | P |  |  |  | P | P | P | P | P | P |  |  |
| *Orchis purpurea* | 9201390 |  |  | P | P | P |  |  |  | P | P | P | P | P | P |  |  |
| *Orchis simia* | 9201391 |  |  | P | P | P |  |  |  | P | P | P | P | P | P |  |  |
| *Orchis ustulata* | 9201392 |  |  | P | P | P |  |  |  | P | P | P | P | P | P |  |  |
| *Origanum vulgare* | 9201393 |  |  | P |  |  |  |  |  | P |  |  |  |  |  |  |  |
| *Ornithopus perpusillus* | 9201397 |  |  | P |  |  |  |  |  | P | P |  |  |  |  |  |  |
| *Parietaria judaica* | 9201435 |  |  | N |  |  |  |  |  | N |  |  |  |  |  |  |  |
| *Parnassia palustris* | 9201437 |  |  | P |  |  |  |  |  | P |  |  |  |  |  |  |  |
| *Pedicularis palustris* | 9201441 |  |  |  |  | P |  |  |  |  |  |  |  | P | P |  |  |
| *Pedicularis sylvatica* | 9201442 |  |  | P |  | P |  |  |  |  | P |  |  | P | P |  |  |
| *Periscaria vivipara* | 9201543 |  |  | P |  |  |  |  |  | P |  |  |  |  |  |  |  |
| *Persicaria bistorta* | 9201525 |  |  |  | P |  |  |  |  |  |  | P | P |  |  |  |  |
| *Phalaris arundinacea* | 9201454 | N | N |  | N | N |  | N | N |  |  | N | N | N | N |  |  |
| *Phleum arenarium* | 9201459 |  |  |  |  |  | P |  |  |  |  |  |  |  |  |  | P |
| *Phleum pratense* | 9202247 |  |  | N | N | N |  |  |  |  | N | N | N | N | N |  |  |
| *Phragmites australis* | 9201465 | N | N |  |  | N | N | N | N |  |  |  |  | N | N | N |  |
| *Picea abies* | 9201470 | N |  |  |  |  | N | N | N |  |  |  |  |  |  | N | N |
| *Picea sitchensis* | 9202401 | N |  |  |  |  | N | N | N |  |  |  |  |  |  | N | N |
| *Pilosella officinarum* | 920976 |  |  | P |  |  |  |  |  | P | P |  |  |  |  |  |  |
| *Pimpinella saxifraga* | 9201476 |  |  | P | P |  |  |  |  | P | P | P | P |  |  |  |  |
| *Pinguicula lusitanica* | 9201480 |  |  |  |  |  | P |  |  |  |  |  |  |  |  | P |  |
| *Pinguicula vulgaris* | 9201481 |  |  | P |  |  | P |  |  | P |  |  |  |  |  | P |  |
| *Pinus sylvestris* | 9201484 | N |  |  |  |  | N | N | N |  |  |  |  |  |  | N | N |
| *Plantago coronopus* | 9201485 |  |  | P |  |  |  |  |  | P | P |  |  |  |  |  |  |
| *Plantago lanceolata* | 9201487 |  |  |  |  |  | P |  |  |  |  |  |  |  |  |  | P |
| *Plantago major* | 9201488 |  |  | N | N |  |  |  |  | N | N | N | N |  |  |  |  |
| *Plantago maritima* | 9201489 |  |  | P |  |  | P |  |  | P |  |  |  |  |  |  | P |
| *Plantago media* | 9201490 |  |  | P |  |  |  |  |  | P |  |  |  |  |  |  |  |
| *Platanthera bifolia* | 9201492 |  |  | P | P | P |  |  |  | P | P | P | P | P | P |  |  |
| *Platanthera chlorantha* | 9201493 |  |  | P | P | P |  |  |  | P | P | P | P | P | P |  |  |
| *Poa trivialis* | 9201507 |  |  |  |  | N |  |  |  |  |  |  |  | N | N |  |  |
| *Polygala amara* | 9201510 |  |  | P | P |  |  |  |  | P | P | P | P |  |  |  |  |
| *Polygala calcarea* | 9201512 |  |  | P | P |  |  |  |  | P | P | P | P |  |  |  |  |
| *Polygala serpylifolia* | 9201514 |  |  | P | P |  | P |  |  | P | P | P | P |  |  | P | P |
| *Polygala vulgaris* | 9201515 |  |  | P | P |  |  |  |  | P | P | P | P |  |  |  |  |
| *Polytrichum alpinum* | 820481 | N |  |  |  |  |  | N | N |  |  |  |  |  |  |  |  |
| *Polytrichum commune* | 820482 | N |  |  |  |  |  | N | N |  |  |  |  |  |  |  |  |
| *Polytrichum formosum* | 820483 | N |  |  |  |  |  | N | N |  |  |  |  |  |  |  |  |
| *Polytrichum juniperinum* | 820485 | N |  |  |  |  |  | N | N |  |  |  |  |  |  |  |  |
| *Polytrichum longisetum* | 820484 | N |  |  |  |  |  | N | N |  |  |  |  |  |  |  |  |
| *Polytrichum piliferum* | 820488 | N |  |  |  |  |  | N | N |  |  |  |  |  |  |  |  |
| *Polytrichum sexangulare* | 820487 | N |  |  |  |  |  | N | N |  |  |  |  |  |  |  |  |
| *Potentilla erecta* | 9201588 |  | P | P | P | P | P |  |  | P | P | P | P | P | P | P | P |
| *Potentilla palustris* | 9201592 |  | P |  | P | P |  |  |  |  |  | P | P | P | P |  |  |
| *Primula farinosa* | 9201603 |  |  | P |  |  |  |  |  | P |  |  |  |  |  |  |  |
| *Primula veris* | 9201605 |  |  | P | P |  |  |  |  | P |  | P | P |  |  |  |  |
| *Prunus spinosa* | 9201617 |  |  | N |  | N | N |  |  | N |  |  |  | N | N | N | N |
| *Pseudorchis albida* | 920947 |  |  | P | P | P |  |  |  | P | P | P | P | P | P |  |  |
| *Pteridium aquilinum* | 9101619 | N | N | N |  | N | N | N | N |  | N |  |  | N | N | N |  |
| *Quercis robur* | 9201640 |  |  |  |  | N | N |  |  |  |  |  |  | N | N | N | N |
| *Quercus petraea* | 9201638 |  |  |  |  | N | N |  |  |  |  |  |  | N | N | N | N |
| *Racomitrium lanuginosum* | 820525 | P |  |  |  |  | P | P | P |  |  |  |  |  |  | P | P |
| *Ranunculus acris* | 9201642 |  |  |  |  |  | N |  |  |  |  |  |  |  |  |  | N |
| *Ranunculus flammula* | 9201651 |  | P |  | P |  | N |  |  |  |  | P | P |  |  |  | N |
| *Ranunculus repens* | 9201660 | N |  | N |  | N | N | N | N |  | N |  |  | N | N | N | N |
| *Rhinanthus minor* | 9201678 |  |  |  | P |  |  |  |  |  |  | P | P |  |  |  |  |
| *Rhododendron ponticum* | 9205194 | N |  | N |  |  | N | N | N |  | N |  |  |  |  |  | N |
| *Rhynchospora alba* | 9201691 | P |  |  |  |  |  | P | P |  |  |  |  |  |  |  |  |
| *Rhynchospora fusca* | 9201692 | P |  |  |  |  |  | P | P |  |  |  |  |  |  |  |  |
| *Rhytidiadelphus squarrosus* | 820533 |  |  | N |  |  |  |  |  |  | N |  |  |  |  |  |  |
| *Rubus chamaemorus* | 9201727 | P |  |  |  |  | P | P | P |  |  |  |  |  |  | P |  |
| *Rubus fruticosus agg.* | 9201728 | N | N |  |  | N | N | N | N |  |  |  |  | N | N | N | N |
| *Rubus idaeus* | 9201729 |  |  |  |  |  | N |  |  |  |  |  |  |  |  | N | N |
| *Rumex acetosa* | 9201734 |  | P |  |  |  |  |  |  |  |  |  |  |  |  |  |  |
| *Rumex acetosella* | 9201735 |  |  | P |  |  | P |  |  | P | P |  |  |  |  |  | P |
| *Rumex crispus* | 9201742 |  |  | N | N | N | N |  |  | N | N | N | N | N | N |  | N |
| *Rumex obtusifolius* | 9201748 |  |  | N | N | N | N |  |  | N | N | N | N | N | N | N | N |
| *Salix alba* | 9201784 | N |  |  |  | N | N | N | N |  |  |  |  | N | N | N |  |
| *Salix arbuscula* | 9201785 | N |  |  |  | N | N | N | N |  |  |  |  | N | N | N |  |
| *Salix aurita* | 9201787 | N |  |  |  | N | N | N | N |  |  |  |  | N | N | N |  |
| *Salix caprea* | 9201788 | N |  |  |  | N | N | N | N |  |  |  |  | N | N | N |  |
| *Salix cinerea* | 9201789 | N |  |  |  | N | N | N | N |  |  |  |  | N | N | N |  |
| *Salix fragilis* | 9201793 | N |  |  |  | N | N | N | N |  |  |  |  | N | N | N |  |
| *Salix herbacea* | 9201794 | N |  |  |  | N | N | N | N |  |  |  |  | N | N | N |  |
| *Salix lanata* | 9201795 | N |  |  |  | N | N | N | N |  |  |  |  | N | N | N |  |
| *Salix lapponum* | 9201796 | N |  |  |  | N | N | N | N |  |  |  |  | N | N | N |  |
| *Salix myrsinifolia* | 9201797 | N |  |  |  | N | N | N | N |  |  |  |  | N | N | N |  |
| *Salix myrsinites* | 9201798 | N |  |  |  | N | N | N | N |  |  |  |  | N | N | N |  |
| *Salix pentandra* | 9201799 | N |  |  |  | N | N | N | N |  |  |  |  | N | N | N |  |
| *Salix phylicifolia* | 9201800 | N |  |  |  | N | N | N | N |  |  |  |  | N | N | N |  |
| *Salix purpurea* | 9201801 | N |  |  |  | N | N | N | N |  |  |  |  | N | N | N |  |
| *Salix repens* | 9201802 |  |  |  |  | P | P |  |  |  |  |  |  | P | P | P | P |
| *Salix reticulata* | 9201803 | N |  | P |  | N | N | N | N | P |  |  |  | N | N | N |  |
| *Salix triandra* | 9201804 | N |  |  |  | N | N | N | N |  |  |  |  | N | N | N |  |
| *Salix viminalis* | 9201805 | N |  |  |  | N | N | N | N |  |  |  |  | N | N | N |  |
| *Sanguisorba minor* | 9205442 |  |  | P | P |  | P |  |  | P |  | P | P |  |  |  | P |
| *Sanguisorba officinalis* | 9201818 |  |  | P | P | P |  |  |  |  | P | P | P | P | P |  |  |
| *Saxifraga aizoides* | 9201826 |  |  | P |  |  |  |  |  | P |  |  |  |  |  |  |  |
| *Saxifraga hypnoides* | 9201835 |  |  | P |  |  |  |  |  | P |  |  |  |  |  |  |  |
| *Saxifraga oppositifolia* | 9201837 |  |  | P |  |  |  |  |  | P |  |  |  |  |  |  |  |
| *Scabiosa columbaria* | 9201846 |  |  | P |  |  |  |  |  | P |  |  |  |  |  |  |  |
| *Schoenus nigricans* | 9201855 |  |  |  |  |  | N |  |  |  |  |  |  |  |  | N |  |
| *Scilla verna* | 9201857 |  |  | P |  |  | P |  |  | P |  |  |  |  |  |  | P |
| *Sedum acre* | 9201875 |  |  | P |  |  | P |  |  | P | P |  |  |  |  |  | P |
| *Sedum album* | 9201876 |  |  | N |  |  |  |  |  | N |  |  |  |  |  |  |  |
| *Sedum anglicum* | 9201877 |  |  | P |  |  |  |  |  | P | P |  |  |  |  |  |  |
| *Senecio aquaticus* | 9201891 |  |  |  |  |  | N |  |  |  |  |  |  |  |  |  | N |
| *Senecio erucifolius* | 9201896 |  |  |  |  |  | N |  |  |  |  |  |  |  |  |  | N |
| *Senecio jacobaea* | 9201899 |  |  | N | N |  | N |  |  | N | N | N | N |  |  | N | N |
| *Senecio vulgaris* | 9201905 |  |  |  |  |  | N |  |  |  |  |  |  |  |  |  | N |
| *Serapias parviflora* | 9204243 |  |  | P | P | P |  |  |  | P | P | P | P | P | P |  |  |
| *Serratula tinctoria* | 9201906 |  |  | P | P | P | P |  |  | P | P | P | P | P | P | P | P |
| *Sesleria caerulea* | 9201908 |  |  | P |  |  |  |  |  | P |  |  |  |  |  |  |  |
| *Sibbaldia procumbens* | 9201913 |  |  | P |  |  |  |  |  | P |  |  |  |  |  |  |  |
| *Silaum silaus* | 9201916 |  |  |  | P |  |  |  |  |  |  | P | P |  |  |  |  |
| *Silene acaulis* | 9201917 |  |  | P |  |  |  |  |  | P |  |  |  |  |  |  |  |
| *Sonchus arvensis* | 9201952 |  |  | N |  |  |  |  |  | N |  |  |  |  |  |  |  |
| *Sonchus asper* | 9201953 |  |  | N |  |  |  |  |  | N |  |  |  |  |  |  |  |
| *Sphagnum auriculatum* | 820578 | P |  |  |  | P | P | P | P |  |  |  |  | P | P | P |  |
| *Sphagnum balticum* | 820552 | P |  |  |  | P | P | P | P |  |  |  |  | P | P | P |  |
| *Sphagnum capillifolium* | 820564 | P |  |  |  | P | P | P | P |  |  |  |  | P | P | P |  |
| *Sphagnum compactum* | 820554 | P |  |  |  | P | P | P | P |  |  |  |  | P | P | P |  |
| *Sphagnum contortum* | 820555 | P |  |  |  | P | P | P | P |  |  |  |  | P | P | P |  |
| *Sphagnum cuspidatum* | 820556 | P | P |  |  | P | P | P | P |  |  |  |  | P | P | P |  |
| *Sphagnum denticulatum* | 820578 |  | P |  |  |  |  |  |  |  |  |  |  |  |  |  |  |
| *Sphagnum fallax* | 820571 |  | P |  |  |  |  |  |  |  |  |  |  |  |  |  |  |
| *Sphagnum fimbriatum* | 820557 | P |  |  |  | P | P | P | P |  |  |  |  | P | P | P |  |
| *Sphagnum fuscum* | 820558 | P |  |  |  | P | P | P | P |  |  |  |  | P | P | P |  |
| *Sphagnum girgensohnii* | 820559 | P |  |  |  | P | P | P | P |  |  |  |  | P | P | P |  |
| *Sphagnum imbricatum* | 820560 | P |  |  |  | P | P | P | P |  |  |  |  | P | P | P |  |
| *Sphagnum lindbergii* | 820561 | P |  |  |  | P | P | P | P |  |  |  |  | P | P | P |  |
| *Sphagnum magellanicum* | 820562 | P |  |  |  | P | P | P | P |  |  |  |  | P | P | P |  |
| *Sphagnum molle* | 820563 | P |  |  |  | P | P | P | P |  |  |  |  | P | P | P |  |
| *Sphagnum palustre* | 820566 | P | P |  |  | P | P | P | P |  |  |  |  | P | P | P |  |
| *Sphagnum papillosum* | 820567 | P |  |  |  | P | P | P | P |  |  |  |  | P | P | P |  |
| *Sphagnum platyphyllum* | 820555.2 | P |  |  |  | P | P | P | P |  |  |  |  | P | P | P |  |
| *Sphagnum pulchrum* | 820569 | P |  |  |  | P | P | P | P |  |  |  |  | P | P | P |  |
| *Sphagnum quinquefarium* | 820570 | P |  |  |  | P | P | P | P |  |  |  |  | P | P | P |  |
| *Sphagnum recurvum* | 820571 |  |  |  |  | P | P |  |  |  |  |  |  | P | P | P |  |
| *Sphagnum riparium* | 820572 | P |  |  |  | P | P | P | P |  |  |  |  | P | P | P |  |
| *Sphagnum russowii* | 820574 | P |  |  |  | P | P | P | P |  |  |  |  | P | P | P |  |
| *Sphagnum squarrosum* | 820575 | P | P |  |  | P | P | P | P |  |  |  |  | P | P | P |  |
| *Sphagnum strictum* | 820576 | P |  |  |  | P | P | P | P |  |  |  |  | P | P | P |  |
| *Sphagnum subnitens* | 820568 | P | P |  |  | P | P | P | P |  |  |  |  | P | P | P |  |
| *Sphagnum tenellum* | 820579 | P |  |  |  | P | P | P | P |  |  |  |  | P | P | P |  |
| *Sphagnum teres* | 820580 | P | P |  |  | P | P | P | P |  |  |  |  | P | P | P |  |
| *Sphagnum warnstorfii* | 820581 | P |  |  |  | P | P | P | P |  |  |  |  | P | P | P |  |
| *Spiranthes aestivalis* | 9201995 |  |  | P | P | P |  |  |  | P | P | P | P | P | P |  |  |
| *Spiranthes romanzoffiana* | 9201996 |  |  | P | P | P |  |  |  | P | P | P | P | P | P |  |  |
| *Spiranthes spiralis* | 9201997 |  |  | P | P | P |  |  |  | P | P | P | P | P | P |  |  |
| *Stachys officinalis* | 920237 |  |  | P | P |  |  |  |  | P | P | P | P |  |  |  |  |
| *Stellaria uliginosa* | 9202007 |  | P |  |  |  |  |  |  |  |  |  |  |  |  |  |  |
| *Succisa pratensis* | 9202021 |  | P | P | P | P | P |  |  | P | P | P | P | P | P | P |  |
| *Teesdalia nudicaulis* | 9202041 |  |  | P |  |  |  |  |  | P |  |  |  |  |  |  |  |
| *Thalictrum alpinum* | 9202047 |  |  | P |  |  |  |  |  | P |  |  |  |  |  |  |  |
| *Thalictrum flavum* | 9202048 |  |  |  | P | P |  |  |  |  |  | P | P | P | P |  |  |
| *Thalictrum minus* | 9202049 |  |  | P |  |  |  |  |  | P |  |  |  |  |  |  |  |
| *Thymus polytrichus* | 9202060 |  |  | P |  |  | P |  |  | P | P |  |  |  |  |  | P |
| *Thymus pulegioides* | 9202061 |  |  | P |  |  |  |  |  | P | P |  |  |  |  |  |  |
| *Trichophorum cespitosum* | 9201858 | P |  |  |  |  | N | P | P |  |  |  |  |  |  |  | N |
| *Trifolium repens* | 9202092 |  |  | N | N | N |  |  |  | N | N | N | N | N | N |  |  |
| *Trinia glauca* | 9202104 |  |  | P |  |  |  |  |  | P |  |  |  |  |  |  |  |
| *Trisetum flavescens* | 9202105 |  |  | N |  |  |  |  |  | N |  |  |  |  |  |  |  |
| *Trollius europaeaus* | 9202106 |  |  |  | P | P |  |  |  |  |  | P | P | P | P |  |  |
| *Typha angustifolia* | 9202110 |  |  |  |  |  | N |  |  |  |  |  |  |  |  | N |  |
| *Typha latifolia* | 9202111 |  | N |  |  |  | N |  |  |  |  |  |  |  |  | N |  |
| *Ulex europaeus* | 9202112 |  |  |  |  | N | N |  |  |  |  |  |  | N | N | N | N |
| *Ulex gallii* | 9202113 |  |  |  |  |  | P |  |  |  |  |  |  |  |  | P | P |
| *Ulex minor* | 9202114 |  |  |  |  |  | P |  |  |  |  |  |  |  |  | P | P |
| *Urtica dioica* | 9202126 | N | N | N | N | N | N | N | N | N | N | N | N | N | N | N | N |
| *Vaccinium myrtillus* | 9202136 | P |  | P |  |  | P | P | P |  | P |  |  |  |  | P | P |
| *Vaccinium oxycoccus* | 9201419 | P |  |  |  |  | P | P | P |  |  |  |  |  |  | P | P |
| *Vaccinium vitis-idaea* | 9202138 | P |  |  |  |  | P | P | P |  |  |  |  |  |  | P | P |
| *Valeriana dioica* | 9202139 |  |  |  | P | P |  |  |  |  |  | P | P | P | P |  |  |
| *Valeriana officinalis* | 9202140 |  |  |  |  | P |  |  |  |  |  |  |  | P | P |  |  |
| *Veronica officinalis* | 9202173 |  |  | P |  |  |  |  |  | P | P |  |  |  |  |  |  |
| *Vicia orobus* | 9202196 |  |  | P |  |  |  |  |  |  | P |  |  |  |  |  |  |
| *Viola hirta* | 9202210 |  |  | P |  |  |  |  |  | P | P |  |  |  |  |  |  |
| *Viola palustris* | 9202215 |  | P | P | P | P |  |  |  |  | P | P | P | P | P |  |  |
| *Viola riviniana* | 9202218 |  |  | P |  |  | P |  |  |  | P |  |  |  |  |  | P |
| *Viola seedling/sp* | 9204565 |  |  | P |  |  |  |  |  |  | P |  |  |  |  |  |  |
