# Supplementary material for: Using Qualitative and Quantitative Methods to Choose a Habitat Quality Metric for Air Pollution Policy Evaluation
Source: PLoS One. 2016 Aug 24;11(8):e0161085. doi: 10.1371/journal.pone.0161085 (PMC4996518; doi:10.1371/journal.pone.0161085)
Supplement: S2 File — (DOCX) [file pone.0161085.s002.docx]

## S2 Extensive summary of interviews

## Topic 1: Main features of habitat quality

#### T1 a) Combination of features

Assessment of habitat quality often involves the consideration a number of different features, including habitat structure and condition, management impacts (for example grazing, drainage or burning) and vegetation characteristics, such as species extent, vegetation composition and structure. For example, water conditions are particularly important for wetlands in addition to vegetation composition:

“*Water quantity and quality is one of the key things. And then vegetation quality…That’s partly species assemblage, but also species of particular note as well, so they’ll come into the factors of selection. Size as well, obviously from the point of view if you’ve got limited resources.” I2 Wetlands [Scotland]*

Another stressed the importance of management and vegetation cover in relation to heathlands:

*“…it would be signs of grazing intensity….How tall, how much dung, how much bare ground… proportion of graminoid species in the habitat, the amount of vegetation being pulled out, and that sort of thing.” I7 Heaths [Wales]*

This combined approach to habitat quality assessment has applicability to all three EUNIS Level 1 habitats considered in this study, although apparently more so for wetlands, and to a lesser extent heathlands, than for grasslands.

#### T1 b) Habitat structure

Although a combination of features may be used in habitat quality assessment, for wetlands, habitat structure was stated as being particularly important. For example, referring to wetlands in Scotland, one specialist stated:

*“…it’s important that you’ve got these building blocks of good quality – in wetlands terms, you’ve got good quality water, you’ve got sufficient water supply to actually maintain the conditions.” I2 Wetlands [Scotland]*

These sentiments were shared by wetland specialists from all four countries of the UK, who expressed the importance of surface topography such as humps, hollows and pools, water conditions, and depth of peat. The impact of management was also highlighted as significantly affecting whether wetlands pass their quality assessment, and indeed often failing due to occurrence of heavy drainage, burning or grazing. However, despite the sometimes poor vegetation quality of many wetlands in England and Wales, the existence of good structural potential often warrants that the site receives conservation attention, for example:

*“It is surprising how often I’ll go to a place and I’ll rate it quite highly and our local officer will say ‘but it doesn’t have any bog moss’ – but ‘yeah, look, it’s five metres of peat in the middle… it’s domed…’” I6 Wetlands [Wales]*

The existence of the structural elements of a wetland habitat, such as deep peat, would also indicate that the habitat should be assessed as a wetland despite what vegetation may be currently growing upon it:

*“So if you’re on deep peat and you’ve got this heathland community… there shouldn’t be a heathland community there should be a bog community. So we do use that to that degree. And if you’re on acid grassland on deep peat, again ... we should be assessing it as degraded bog. And management should be moving towards getting back to a less degraded bog.” I11a Heaths, Wetlands, Grasslands [England]*

Structural elements can therefore define the habitat classification, regardless of vegetation composition. Evidently habitat structure is crucial for wetlands, but one respondent did also draw attention to its increasing relevance for grassland habitats, especially under changing climatic conditions:

*“This year I’ve seen a lot of bare ground caused by poaching by the very cold winter and cold spring. So I wouldn’t like to suggest that structure is not an important aspect of that assessment…. It seems that this is an exceptional year, an exceptional problem of an exceptional year, but given that we must expect more exceptional years, it is something that we need to address.” I1 Grasslands [Scotland]*

#### T1 c) Vegetation composition and structure

Vegetation, both in terms of composition and structure, emerged as the dominant factor in habitat quality assessment for grasslands and heathlands. The National Vegetation Classification (NVC) framework is often used as a starting point in quality assessment and for site designation:

*“We rely quite heavily on the National Vegetation Classification framework… So, all of our SSSIs now, and have been for some time, unpinned by a survey of that level of detail, an NVC survey.” I10 Grasslands [England]*

Beyond the NVC classification, assessment of the habitat vegetation can involve a number of deliberations, such as how the presence or absence of species may reflect the impacts, site condition, or conservation value, as expressed by one grassland specialist in Wales:

*“It [the NVC community] is the first thing I’d home in on. But then you’d be thinking, is it good for this, is it good for that? And you wanted to know why, is it modified by enrichment or something, in which case you’d mark it down. And you’d be doing that largely on species presence or absence. Or is it transitional to something else? Or is it something completely unique that we’ve not seen before, but still has a diverse species component? So it’s a guide to get us started, but it’s not the final word in conservation assessment by any means.” I8 Grasslands [Wales]*

Generally, species assemblages were considered more important for habitat quality assessment than individual species, because assemblages may be more representative and resilient, and also because most habitats do not necessarily rely on individual species for their functioning:

*“Probably I would be less inclined in notifying individual species of importance, but a range of species, so I’m quite keen on species assemblages, because I think … it’s more representative, and it’s also more resilient to change.” I2 Wetlands [Scotland]*

And similarly:

*“Generally [we’re] not looking for specific species, looking more for diversity of a certain level… you can set site-specific requirements for particular species, but there aren’t that many sites that are dependent on single species” I1 Grasslands [Scotland]*

However, both individual species and species-assemblages or groups of species can be useful for assessing habitat quality if they act as a proxy for environmental conditions, by for example providing some indication of water quality and quantity, nutrient loading or management such as burning:

*“In most circumstances, if I’m looking at habitat quality, I would be looking at individual species or groups of individual species with … environmental requirements as some kind of proxy of the environmental conditions.” I12 Wetlands [England]*

Given that species assemblages are important, a further consideration is how to measure these assemblages. Specialists of both grasslands and heathlands highlighted that species richness would not be a suitable metric for measuring quality in these habitats, due to the fact that the habitats can be naturally species-poor yet still of high quality:

*“…heathlands in many cases are a species-poor habitat, so* *if you increase the number of species, the species richness, it usually means you are increasing the number of generalists, and usually this is a sign of neglect or unfit management.* *So if you increase the number of species by increasing bramble, bracken, ragwort or other species, you may be increasing the number of species but not the heathland species. So species richness is not a sign of quality.” I13 Heaths [England]*

Another aspect to vegetation assessment is the relative importance of vegetation composition compared to vegetation structure. Although composition may be the dominant criterion, vegetation structure is also important as a habitat quality criterion:

*“Vegetation composition and vegetation structure, I think those would be the most important. So, if we were assessing, we would probably start with a Phase 2 type survey and look at composition, but then we would also look the assessment of the structure of the vegetation as well…. I’d say they are equally important. I think, there has been a tendency to focus on the composition, but I think we are more and more thinking that structure is equally important in terms of heathland species, and association with particular structures, structure of the vegetation.” I5 Heaths [Wales]*

#### T1 d) Geographical and temporal variability

When making an assessment about habitat quality, a number of respondents emphasised the need to put the assessment into geographical context. This reflects the fact that often different parts of the country or different altitudes will have natural variations in quality (whether vegetation or structure). The habitat may therefore be assessed with this in mind, although it may not necessarily be a dominating factor in quality assessment. For example:

*“In terms of the community structure within the wider habitat, that sort of thing can be very variable, depending on the where you are in the country, so you’ve got longitudinal and latitudinal variation, but also altitudinal variation”* *I3 Wetlands [Scotland]*

*“So what you regard as high quality in the north of Scotland, where there’s a much smaller pool of species, might be a bit different from the south of Scotland. But we try not to skew our ideas of quality too much.” I1 Grasslands [Scotland]*

However, in some cases, comparison of sites in different parts of the country in terms of quality (for example for SSSI designation) may no longer even be possible due to the rarity of the habitat, and instead a ‘minimum standards approach’ adopted:

*“It all comes down to whether we take a kind of minimum standards approach or an exemplar approach. So those very rare habitats, provided that the grassland comes out as one of the unimproved types, then we wouldn’t start to distinguish between say, an example in say Northeast Derbyshire and one in South Derbyshire and try and compare them*…..*grasslands are so rare now that for most of them we tend to take a minimum standards approach.” I10 Grasslands [England*]

Related to geographical variation, it was also noted that some site have historical anthropogenic impacts that affect quality, which also may be factored in during site assessment:

*“The problem with English blanket bogs is that there’s a much greater history of intensive use, in some cases in the South Pennines going back a thousand years. And that use is basically grazing, historic wildfires have been a problem, and then rotational burning, for grouse and sheep management.” I11b Wetlands [England]*

Just as a habitat may vary naturally depending on geographical factors, a habitat may also vary through time, showing natural dynamism. During habitat quality assessment, dynamism within a habitat may be allowed for, rather than achieving a fixed target of species composition and structure. For example, in relation to wetlands in Scotland, one respondent stated:

*“…one of the things we’re doing, all the time we’re doing, is just taking snap shots, snap shots, snap shots, and sometimes, particularly the wetlands, they are very dynamic, if we let them be dynamic. And I’ve seen it where at certain stages you’ll see something dominant, and then all of sudden it starts to disappear or decline …*

*… the way we look is not flexible enough, it’s too rigid, it’s not dynamic. Habitats are dynamic.” I2 Wetlands [Scotland]*

#### T1 e) Ecosystem services

Since the ecosystem services framework is currently favoured as an approach to supporting land management decisions, it is feasible that the capability of a habitat to deliver multiple ecosystem services such as climate or water regulation in addition to wild species diversity may be considered within habitat quality assessments. Ecosystem services did not emerge as a dominant theme from the consultations. However, there was some opinion that ecosystem services could be included in habitat quality assessment as an additional factor, or that the current approach already fits in fairly effectively to the ecosystem services framework:

*“I think blanket bog fits quite well with ecosystem services… but notifying land as nature reserves, then obviously they need to be important for nature, if they are for ecosystem services then they should be called ecosystem reserves rather than if their biodiversity isn’t… but there’s not generally too much of a conflict in priorities… or there shouldn’t be. Apart from perhaps recreation might be one… we work very closely with the water companies in the uplands in terms of producing good quality water, protecting carbon…” I11a Wetlands [England]*

*“I’m not saying that these systems couldn’t be valued using ecosystem service provision, but so far we haven’t gone down that route. I’d see they’d have to be an add on, it wouldn’t be an either/or, would it?”* *I10 Grasslands [England]*

#### T1 f) Applicability and practicality

The Common Standards Monitoring (CSM) guidance appeared to be widely used as the fundamental guideline for the monitoring the vegetation features of habitat quality. The use of the CSM guidance was raised by specialists from all habitats across all four countries, and in general the specialists considered that the guidance was useful and appropriate. Several specialists referred to their involvement in drawing up the CSM guidance and indicator-species lists.

*“I have found that, yes there are lots of things that you can criticise about Common Standards Monitoring, there always will be some things because it is so vast, we’re trying to do such a vast job, but I think the approach, I think is good. It does give us a standardisation which allows us to make judgements in terms of a habitat at the level of Scotland…which I think gives us a good level of standardisation for some of our habitats … So I think that approach serves us well*.” *I4 Heaths, Wetlands, Grasslands [Scotland]*

However, a common issue arising was that CSM, by attempting to be applicable to the whole of the UK, can be too generic and broad:

*“…one of the issues with Common Standards Monitoring is that we’ve tried to include all four countries within one set of guidances, and for certain species it is not always appropriate for your location. But I would have thought it covers most of what we’d consider.” I5 Heaths [Wales]*

As a consequence of the necessarily broad nature of CSM, the majority of specialists had modified indicator-species lists to improve their applicability to their country, to particular areas or even to individual sites. This includes removing species that are not considered applicable, and adding additional ones, for example ones that are locally distinctive. This can be useful for detecting local changes and potential negative impacts in an area, as explained below:

*“On specific sites there may be more monitoring. On those sites that are very rich we’d expect the advisor to include a list of species that you would look for in subsequent years. Those species would not be listed on the generic UK form but would be added for that particular site to emphasise its species richness; and when they visited the site in six years time if they could detect obvious change then they should try and work out, why is that? And if the grazing seems to be okay and the burning is in hand, there’s no drainage, then… that only leaves N deposition!” I11a Heaths, Wetlands, Grasslands [England]*

A key consideration in drawing up the indicator-species lists for CSM guidance was practicality, i.e. whether species were easily observed and identified by surveyors without specialist knowledge:

*“We tried also to concentrate on species that are easy to identify. So we tried to avoid, where possible, grasses and sedges – not always, we did use them sometimes. And we also tried to use species that were visible for a reasonable time during the season – you know things that appear and disappear very quickly are not so useful, because bear in mind the level of competency of the people who might be doing this.” I10 Grasslands [England]*

Even so, some specialists reported tailoring the lists to make them more practical for the local officers to carry out the monitoring, restricted by limitations in skill, experience or time. For example:

*“In grasslands it [the modified indicator-species list] was developed to have generic attributes that are easy to apply to a site. This was so that the average Area Officer could go out and do it without specialist skills.” I1 Grasslands [Scotland]*

Furthermore, the formal guidelines are not necessarily the only way that the vegetation should be assessed, and there was some indication that local officers undertaking the habitat assessments should ideally being using judgement as well. However, lack of experience was cited as problem with this, as expressed below:

*“I always, say ‘use your judgement as much as possible’ but people on the whole don’t feel able to use their judgement. It’s the same in England. You’ve got people who haven’t worked on the habitat enough to feel that they can apply judgement. And so they tend to stick to the letter of the guidance rather too much, but it’s never that clear-cut, is it?”* *I1 Grasslands [Scotland]*

## Topic 2: Value of individual species

#### T2 a) Structural and functional species

Specialists concerned with all three EUNIS Level 1 habitats considered in this study highlight the importance of species that are structurally or functionally significant. These species seem particularly important for wetland habitats, for example species that help to form peat and maintain the overall functionality:

*“The other types of species that you could look at in terms of habitats like bogs for example, are the ones that are structurally important, or functionally important, peat forming species, the* Sphagna *and the* Eriophorum *in particular, could be ones that we would view of being of particular value and being particularly important in those habitats.” I4 Wetlands [Scotland]*

It was also noted by some specialists that functional species are often interchangeable, and the specific genus or species may not be important:

*“If all the* Sphagnum *was to go, that’s potentially more significant. But bogs in the northwest often don’t have Sphagna. What tends to happen is that* Sphagnum *is replaced by* Racomitrium*, and in the northwest by* Campylopus atrovirens*, which again seems to fulfil a similar role to what* Sphagna *does. It’s not such a prolific peat former but it does form peat, it does help cover the surface. It impedes water flow… although if you were to lose your* Sphagna *in the east, you wouldn’t get* Racomitrium *coming in. They are more equal in some places than others.” I3 Wetlands [Scotland]*

Functional species are also important for other habitats, particularly if they help to maintain system resilience in the face of changing climate, as illustrated below with respect to grasslands:

*“Particularly with climate change, we have to consider, what’s their role in the resilience of that. They may not be the rarest but they may have a key function in the way that habitat functions and the stability of that habitat. And those things, we don’t necessarily explore when it’s sort of in bright lights that you must protect this species because it is rare.” I8 Grasslands [Wales]*

#### T2 b) Scarce species

The general consensus was that scarce or rare species provide added value to a habitat, but are not usually a dominant criterion for assessing habitat quality, for example:

*“I think that the mega-rare things are just used as a bonus evaluation. A site will stand on its own two feet because of the distinctive elements of it... I can’t think of many terrestrial wetland sites where rare species are the main decisive factor, I can’t think of any actually. It tends to be much more on the habitat intactness or assemblage of characteristics, even if they might actually be becoming rare.” I6 Wetlands [Wales]*

However, there may be some circumstances where a rare or scarce species may be the defining part feature of a habitat, such as some montane habitats:

*“Well there are some habitats where it’s really the scarce species that are effectively the defining part of the habitat, so in montane willow scrub there are half a dozen rare dwarf willow species that are what basically what makes the habitat what it is.” I4 Heaths, Wetlands, Grasslands [Scotland]*

Scarcity or rarity of a species depends on scale – whether the species is being considered with respect to local, national or international abundance and distribution. The reference scale used is likely to vary depending on context, however one specialist indicated that UK distribution was of particular importance:

*“You can look at different scales for different purposes. I work on a Scottish remit so I am looking at .... Something that’s uncommon down in the south, that adds value…possibly the UK distribution is more important than the Scottish.” I1 Grasslands [Scotland]*

Scarce species may also be important for site designation, even if they are not an important part of the general habitat quality assessment:

*“It [the presence of nationally scarce species] would carry a fair bit of weight if you were choosing to designate a particular site. I suspect it doesn’t carry as much weight under our monitoring methodology.” I1 Grasslands [Scotland]*

A number of specialists also emphasised that using scarce species as a means of assessing habitat quality is not appropriate. This may be because some scarce species have become so rare that they no longer provide useful information for quality assessment, but also because a scarce species may not be critical for the habitat functioning:

*“The thing is, they are so scarce now that they can’t really tell us a lot about our heathland because they no longer have those species. So scarce species may be important in a local context, where you know that that species still exists and is and should be there. But I don’t know if you can use them across Wales, because so many parts of Wales now, as I say if you go to the Lleyn, there’s only one or two sites which have some of these scarce species. So, that’s why I don’t know how you would use them more broadly” I5 Heaths [Wales]*

*“If we take Dwarf birch,* Betula nana*…It’s a nationally scarce species. If the* Betula nana *was to disappear or shrink its range, it’s not going to affect the bog’s capacity to do what the bog does. It’s still going to capture carbon, it’s still going to moderate water flows, all other things being equal – it’s just the fact that that species has gone. So in that respect you could say that it’s not that important for the habitat.” I3 Wetlands [Scotland]*

#### T2 c) Invasive species

The prevailing attitude of the habitat specialists, from all habitat types, was that invasive species are not negative *per se*, i.e. not intrinsically negative, but rather they would usually only be considered negative if they caused some detrimental impact, usually through out-competing native species that are considered desirable. For example, one respondent stated:

*“If you’ve got something that’s invasive, even if it’s a non-native, but it’s not actually affecting the species composition otherwise, then I don’t think you’d worry too much about it. It’s something you’d note down obviously, and you wouldn’t want it to increase...but if it’s just there at low cover then so what? But when it builds in cover and is affecting other species, that’s entirely different.” I8 Grasslands [Wales]*

Nevertheless, there was some suggestion that alien (non-native) species are generically negative and should be eradicated if possible, although this sentiment may be in part a reflection of their experience of particularly detrimental species such as *Rhododendron,* which can be a serious, although localised, problem on heaths and wetlands:

*“We tend to say that the exotics, we should try to eradicate if possible. So for example, for the heathlands it’s* Rhododendron*,* Gaultheria*, those are the main species that cause problems, and they tend to spread and again out-compete the characteristic species, so if possible, less than 1% and if possible eradicated. Whereas the natives, we wouldn’t say eradicate.” I13 Heaths [England]*

Another factor that appears to be important with regards to invasive species is the feasibility of being able to remove or control the species. In cases where the invading species is so widespread that removing them it is not pragmatic, it was suggested that this may contribute to a more accepting view of the species, particularly if the impacts of the species are not well known – for example the New Zealand willowherb *(Epilobium brunnescens)* in upland habitats:

*“Yes, I think it would be nice if we didn’t have non-native species where we don’t want them. If they are not causing damage to the habitat, which is not always easy to know, do you need to do anything about it? And I think if we can, and we can really easily then we should, but, in the example of the New Zealand willowherb, you wouldn’t have a hope to do it....*

*...do we actually know that it’s having a negative impact, in that, is it actually occupying ground that would otherwise be occupied by other species?” I4 Heaths, Wetlands, Grasslands [Scotland]*

Invasion that appears to be part of a natural change in species range may be considered as neutral or positive, as they form part of natural habitat dynamism, for example the possible invasion of the tongue orchid to the south coast of England:

*“We already get tongue orchid on the south coast, and nobody’s really sure ....the seeds could have just blown across the channel, and if climate change has created the conditions that makes it more suitable, I’d say in that case, that’s fine. That’s part of dynamism, isn’t it really? It’s rather a different situation to your Himalayan balsam I guess, isn’t it?” I10 Grasslands [England]*

#### T2 d) Historical context

The historical context of a site can be relevant when considering which species are of value to a habitat. For example, it may be important to define an appropriate historical reference point to use as a management goal – failure to do so may result in valuing species which historically were not present on that habitat, as explained below in the context of wetlands:

*“The local importance is relevant, but you have to be careful that what you are seeing there that’s local is representative of what you’re actually trying to protect, because it could be what you see there now is not what was there 50 years ago, or the real function. It could be, for example, a swamp may be covered in reed sweet-grass, and that would be a response to the nutrients coming in to the site, and 50 years ago it wouldn’t have even be recorded there. So you have to be careful that we’re not automatically considering that reed sweet-grass is very good...” I2 Wetlands [Scotland]*

A similar issue was highlighted with respect to acid heathlands, which are particularly species-poor. This has resulted in debate over whether this paucity of species is natural or a result of past management, and as such, how it should influence management goals:

*“It’s the bulk of the heaths that are the acid heaths which can be incredibly species poor, and at the back of your mind you think ‘are they species-poor because of past management or is that the way that they naturally are’? And we have this discussion constantly about acid heaths, because some of them are incredibly species-poor. But, our feeling is that maybe historically they weren’t as species-poor as they are now, I think it’s a combination of factors which has led to a decline of these associated species.” I5 Heaths [Wales]*

#### T2 e) Comparative values of species

The valuing of some species over others is a potentially difficult issue. Although conflict did not emerge as a significant problem with respect to plant species valuation, in some cases such issues do arise. For example, preferences of the wider public may not reflect preferences of conservationists, or conflict may arise in the conservation of different scarce species that require conflicting management regimes, for example:

*“.. a site that’s got globe flower, which quite likes grazing, and at the same time it’s got, in the same grazing unit, it’s got Scottish primrose.. It’s how you arrive at a grazing regime that keeps both… So it’s difficult to actually say ‘no, we’re abandoning the globe flower for the Scottish primrose’. You can’t really say that.” I2 Grasslands [Scotland]*

## Topic 3: Plant & lichen indicator-species

#### T3 a) Characteristics of positive indicator-species

There are number of possible characteristics of positive plant and lichen indicator species. Specialists of both wetland and grassland habitats suggested that positive indicators may be distinctive species that are indicative of that particular habitat, for example those that indicate the presence of deep peat in wetlands such as *Eriophorum vaginatum*, or forb species which are unique to grassland sub-communities:

*“…there are species that are indicative of particular habitats. If you think about a species that could be found in a range of habitats, so if what you are interested in is particular habitats, then the species that are indicative of those habitats are going to be the ones that you will use to value that habitat. If you think of grasslands, you get the same, or some of the same grass species,* fescues *and* Agrostises*, you’ll get them both in acid grasslands and in calcareous grasslands, and it’s the forbs that accompany them that are indicative of whether it’s an acid grassland or a calcareous grassland. So, your method with the grassland, you would look at the forb species as ones that are indicative of that habitat.” I4 Grasslands [Scotland]*

Positive indicators may also be species that are typical or common in the habitat. These are likely to be dominant for the habitat, and other accompanying species may be expected throughout the vegetation for it to be considered of high quality. This approach appears particularly useful for heathlands, as illustrated below with respect to montane heaths:

*“It’s the quantity [of* Racomitrium*] that you’re really interested in, it’s the dominant species for that, or most of the forms of that habitat – not all of them.…. It needs other species on top of* Racomitrium*, I can think of about half a dozen species which would be useful to have... like stiff sedge,* Carex bigelowii, *and perhaps dwarf willow and species like that, and a range of lichens as well,* Cetraria islandica*,* Cladonia arbuscula*. I think you want them not necessarily in high cover but frequent occurrence throughout the vegetation.” I7 Heaths [Wales]*

Another characteristic of positive indicators is that they can act as a proxy for good environmental conditions, for example, in wetlands, certain species may indicate high water levels or low nutrient levels:

*“So in a bog it [useful indicators] would be something like* Sphagnum capillifolium*… not the more nutrient-responding species like* S. fallax *or some of the others. Or those that are indicative of long-term stability and clean water, high water levels. And equally in a more alkaline fen, again you’re looking at bryophytes characteristic of high, constant flushing. And low nutrient status. So a lot of the curly brown mosses. And sedges that are indicative of low nutrient status and permanently high water tables.” I12 Wetlands [England]*

*“So I think you need to choose species that are sensitive to perturbation in some way, whether it be atmospheric pollution, or intolerance of some other factor.”*  *I10 Grasslands [England]*

It should be noted, however, that the species-indicators of environmental conditions that were included in the CSM guidance do not include those indicating atmospheric N pollution, since these have only recently become available ([Stevens et al., 2009](#_ENREF_26)).

Furthermore, positive indicators in otherwise poor quality habitat can indicate restoration potential, which may be especially relevant for more the rare, or nationally declining, habitats, such as some grasslands:

*“I know that when the grey dune becomes more and more grass-dominated, you tend to lose most of the species, but a couple of things like* Galium verum *and probably* Lotus corniculatus *will hang on. But that isn’t the same as saying they aren’t good indicators because … even those guys that will hang on still give you an indication that if you can get the management reversed and back in the right direction then that’s probably going to be more restorable or restorable more quickly than something that doesn’t have any of the indicators left.” I9 Grasslands [Northern Ireland]*

#### T3 b) Characteristics of negative indicator-species

Negative indicators are typically those that out-compete other species (such as positive indicators and other valued species) in terms of physical space and proportion of cover, and therefore may often be invasive species (either native or non-native). For wetlands, this may include high proportions of *Molinia.* For heaths, invasive species such as *Rhododendron ponticum* are problematic, or too much cover of *Nardus*, *Juncus squarrosus, Juncus effusus*, *Deschampsia* and *Molinia* as well as non-native trees such as conifers. For grasslands, competitive negative species include species such as *Holcus* and bracken (as stated below):

*“The worst negative indicators are the ones that take up most space. So bracken is probably the worst, just because it reduces the extent of the species-rich grassland. And then species that react to high nutrient levels – if you see lots of* Holcus *it’s a bad sign. Again it’s taking up a lot of space at the expense of other things. The interesting species probably like nitrogen as well but* Holcus *outcompetes them. So it’s species that take up space at the expense of a greater variety of non-competitive things.” I1 Grasslands [Scotland]*

Just as some positive indicators act as a proxy for good environmental conditions, similarly negative indicators can act as a proxy for poor conditions, such as heavy grazing, eutrophication, and the amount of cover, which are important considerations for grassland habitats:

*“So some of the indicators are indicating that it’s an open community and some are indicating that it’s a more closed community. …You’ve also got the element of different management impacts and how they might be reflected in the species composition. So presence or absence of some of those indicators might indicate lack of grazing, or overgrazing. Some of them might indicate things like eutrophication. So the list is based on several factors that might indicate the condition of the habitat.” I9 Grasslands [Northern Ireland]*

In some circumstances, ecosystem service provision may affect the choice of indicator species. For example, species that have been considered as positive indicators may have negative impacts on ecosystem service delivery such as climate regulation. Such a trade-off has been suggested for cotton-grass (*Eriophorum vaginatum)*, which is suspected of increasing methane emissions from wetland habitats, at least in some stages of its growth. Such as trade-off may not necessitate ‘down-grading’ the species to a negative indicator, but may be a consideration in habitat quality assessment:

“*Well – I used to think so [that* Eriophorum vaginatum *is a positive indicator]. My slight hesitation is because it,* Eriophorum vaginatum*, is one of these species that transports methane to the atmosphere. So the fact that we know that it’s shunting all this methane up into at the moment is maybe not quite so good.” I3 Wetlands [Scotland]*

*“Cotton-grass is still a peat-forming species, so you need to look at the balances. …. There may be a trade-off there [between cotton-grass and methane emissions] but at the moment I’m happier seeing cotton-grass on a bog than having it completely absent.” I11b Wetlands [England]*

#### T3 c) Context of indicator-species

Although general characteristics of positive and negative indicators may be identified, a common theme expressed by the habitat specialists was that species indicators are very much context-dependent. For example, differences in location and altitude, soil type or past management may affect what would be expected, desirable or undesirable at a site, as illustrated below with respect to altitude of heathlands:

*“I suppose compositions, even ericoid composition, is different between upland and lowland. We don’t get* Vaccinium *in the lowlands for example, but it’s a key component in the uplands, those kind of issues. And does it make a difference – much of our upland is shallow peaty type soils whereas our lowlands can be quite leachy soils.” I5 Heaths [Wales]*

Natural variability in habitats and sub-communities can also make it difficult to determine which species may be important in terms of function and structure:

“*When you start looking at fens, swamp and marsh… they can be broken down into more different types, they’ve each got their individuality ....It’s hard to define which of those other species are actually important for that habitat to actually function, what their role is, and the importance of that role.” I2 Wetlands [Scotland]*

Indicator species can also change from being positive to being or negative if they become very abundant and outcompete other species. In some examples of habitats, positive indicator-species may not occur, which can make habitat quality assessment difficult when other attributes suggest favourable condition:

*“And so there’s balance of negative species, they tend to be… they’re interchangeable, they can be positive one minute and when they get to a certain state they become negative. So it’s quite difficult.” I2 Wetlands [Scotland]*

*“But what do you do if you don’t find* Rhynchospora*, when you’re obviously in a nice, wet blanket bog? And sometimes you find it in places where you think, there’s a raised bog just near here, and* Rhynchospora *comes out up there, it just doesn’t seem the right conditions.” I2 Wetlands [Scotland]*

The scale at which management and habitat quality assessment is made is may also be an issue with respect to species-indicators, as one specialist raised concern over an increasing trend to micro-manage, rather than considering the ecosystem as a whole.

## Topic 4: Taxa other than plants and lichens

#### T4 a) Importance of other taxa

Other taxa were generally considered to have some importance for a habitat, and one specialist stated that they are an integral attribute:

***“****The quality of the habitat has to include fauna as well... you can’t disassociate the two. They are an integral part.... It makes life easier if we do separate it down, but I don’t think it’s representative.” I2 Wetlands [Scotland]*

However, the general message emerging was that plant and lichen species are most important for the assessment of habitat quality, but other taxa may be assessed in certain circumstances, particularly if the site has been designated based on the presence of other taxa:

*“Well, our condition assessment will be features-based, so we’re looking at the features, so if the site’s declared as an example of grey dune, the assessment of grey dune is based on the plants essentially, but obviously the site could also be declared as an invertebrate assemblage, in which an independent assessment of the invertebrate assemblage would also be carried out. So it’s very much based on the feature, and if the feature is a habitat, then the habitat assessment is largely based on the plants.” I9 Grasslands [Northern Ireland]*

Typically, other taxa will be assessed by specialists in those taxa, rather than the habitat specialists or officers, although some informal assessment may be carried out as part of the general habitat quality assessment, as explained below:

*“I think we would look at the Section 42 species and we would definitely look at those [other taxa] in the context of evaluating, and there are certain species that have a close association with heathland, which we do look at, and we do consider. So things like silver-studded blue butterfly, those kind of things. For the lowlands and the coastal heaths, choughs are very important. So they are key in our assessment of heathlands and getting that balance between what choughs like and what we want from a heathland. So we definitely do look at them. But I don’t think we have any systematic way of assessing, because Common Standards doesn’t really cover the associated non-plant species very well. It’s more a tick list I suppose. You know, this is a silver-studded blue site therefore it is important, rather than having an actual mechanism for grading sites based on their invertebrates or birds.” I5 Heaths [Wales]*

#### T4 b) Management conflicts

Conservation of other taxa can lead to conflict or tension when their habitat requirements or impacts do not coincide with a high quality habitat from a floristic perspective. This emerged as a potential issue across all three habitats. Examples include golden plover (which require bare peat in wetlands), red deer (which can negatively impact on vegetation through herbivory and trampling), some butterflies (e.g. those that require scrub in grasslands) and potentially some lizards (which require bare ground on heaths). Such tensions were thought to be greater at small sites, where there is less opportunity to vary management across the site for multiple purposes. However, in general it was considered that the differences in management needs of other taxa and the vegetation could be accommodated, particularly over larger sites, as explained below:

*“... it’s odd because some of the birds that are prized from the conservation viewpoint are actually associated with degraded sites. So golden plover is the best example, it seems to like very ultra-short vegetation and patches of bare peat, because it can spot predators coming….There is that ability in the Common Standards Monitoring to add certain indicators of local distinctness, and I think you’d have to come to some intelligent decision on a big upland peat massif, along the lines of well 5% for golden plover of trashed bog is a good thing, rather than 100% favourable condition floristically, because that would reduce the golden plover.” I6 Wetlands [Wales]*

*“And managing for marsh fritillary, you’re managing for the habitat conservation. So usually the two are compatible but occasionally you do get conflicts. On some of the small sites particularly.” I8 Grasslands [Wales]*

#### T4 c) Barriers to using other taxa

There are evidently several barriers to using other taxa for assessing habitat quality, which to some extent may contribute to the focus on vegetation in habitat quality assessments. A key barrier, applicable to all habitat and countries, are the limitations in resources, time, and skill of the local officers:

*“We don’t use other groups, because we don’t have the manpower. With plants the Area Officers can be taught to go out and identify the main species, but with invertebrates you couldn’t do that, apart from the obvious groups like butterflies. They are not good as practical indicators in the field. So you can’t even use fungi, for example, they remain a specialist study.” I1 Grasslands [Scotland]*

Furthermore, there is also the potential problem of consistency when using other taxa, as most fauna are not reliably visible – for example sighting butterflies in poor weather conditions, or nesting birds:

*“...they make their nest in the moss, and are very well camouflaged. So if you’ve got dotterel nesting up there then you might think that it’s better quality. But the chances of seeing them are fairly low.” I7 Heaths [Wales]*

*“...the species might not be obvious or it might not be the right time of year” I4 Heaths, Wetlands, Grassland [Scotland]*

In addition to these practical barriers, it was also argued that lack of understanding of the autecology of other taxa makes them less useful in habitat quality assessment:

*“... what isn’t quite known is whether there is enough information on the species’ relationship to vegetation … and whether they really indicate quality or not... I think whether the inverts add any further quality assessment value over and above the floristics, I don’t know. I don’t know anybody that does it actually.” I6 Wetlands [Wales]*

#### T4 d) Proxy indicators of suitability for other taxa

Given the barriers to using other taxa in habitat quality assessments, other taxa are (or could be) assessed using vegetation composition or habitat structure as a proxy for monitoring their populations. There are limitations with this approach:

*“To a certain extent, assessment for other groups has tended to rely on surrogate measures to a certain extent, hasn’t it? But the problem is, if you just took my plant community condition assessment for grasslands, the problem with that is that it doesn’t pick up some of the other structures that might be required for say invertebrates, does it?.” I10 Grasslands [England]*

Therefore, assessing aspects of habitat structure that are not directly related to vegetation composition may be more effective than using vegetation composition alone:

*“... if you’ve got an invertebrate that depends on dead wood, and your site is important for that invertebrate, then you can either go and check if the invertebrate’s there or you could go and check if there is dead wood.” I4 Heaths, Wetlands, Grasslands [Scotland]*

*“One of the things we’ve been doing... is actually looking at micro-niches in habitats, and looking at the suite of species that use those. And that’s particularly important for heathlands. So, which of the species need bare ground, for example, which of the species needs tall heather, which need short heather...” I5 Heaths [Wales]*

## Topic 5: Species-groups

#### T5 a) Pros and cons of using species-groups

Assessing cover of species-groups, sometimes in proportion to each other, has some potential benefits as a measure of habitat quality in all three habitats. Proportion cover can provide a useful guidance on the general condition of a habitat, from which management requirements can be inferred:

*“It sets limits to say if you are growing too far in one direction, then perhaps you need to consider the habitat.” I4 Heaths [Scotland]*

Using estimates of species group cover can also be useful for verifying the accuracy of estimations of individual species cover:

*“Both on heaths and on bogs we would record dwarf shrub cover, grass cover, bryophyte cover, lichen cover, those sorts of broad headings. But we would also record the individual dwarf shrubs themselves... So it’s actually quite a useful check that you’ve made your original estimation quite good” I9 Wetlands, Heaths [Northern Ireland]*

However, using species groups can depend on the interpretation of habitat quality, and may have more relevance when considering other ecosystem services, rather than specifically biodiversity, as explained below:

*“If you’re wanting the bog to capture carbon, or to moderate water flows, or provide grazing for sheep, deer, then the actual individual species don’t matter too much. … It does make a difference if you’re looking at it from a biodiversity perspective – do you still have your cranberries, sundews, etc. It depends what services you want your bog to provide.” I3 Wetlands [Scotland]*

Furthermore, in some cases – such as for the more scarce sub-communities – assessment at the species level is more useful than assessing species-groups, as species-groups do not provide the level of detail necessary to gauge habitat quality:

*“...there’s certain groups like dwarf shrubs that are useful for most examples. But if there are specific sub-types of habitats, which are often ones that are more scarce, then we do need to determine at the deeper, species level.” I4 Heaths [Scotland]*

A species-group level of assessment may also not provide sufficient information about environmental conditions, as individual species within a group may respond differently to different environmental factors, and some species within a group may not be considered a positive indicator in the same way as the others:

*“It’s more on the ecology of the* Sphagna *so if some of the* Sphagna *indicate something damaging on the site like water movement where you wouldn’t expect water movement or enrichment where you wouldn’t expect enrichment then they wouldn’t count. So something like* S. squarrosum *on a bog is something that you wouldn’t want to see on a bog. You’d see it around the edges of a bog but you wouldn’t want to see it on the main surface, on the intact surface of the bog. Something like that we’d record, well we wouldn’t record it as a negative but … we wouldn’t include it as part of the suite of* Sphagnum *that we’re trying to estimate cover for.” I9 Wetlands [Northern Ireland]*

#### T5 b) Identifying useful species-groups

The percentage cover of forbs or herbs, which may typically be considered positive indicators, can be useful for assessing the quality of some types of grasslands, particularly for cases that are borderline fail:

*“Well percent forb cover is one of the main attributes – in other words the feature could pass or fail on the basis of that whereas the structural attributes don’t affect a pass or fail, so it is important. It’s only used for neutral and calcareous grassland because it is just too difficult to assess forb cover where there are lots of bushes. So yes I think it is important, but it tends to just back up what you’ve already discovered, which is fair enough – if you’ve already spotted lots of important indicators, you might not rely on forb cover. It’s important if you think you’ve got a borderline case, if you are not sure whether to call it favourable or unfavourable, it does help with that.” I1 Grasslands [Scotland]*

*“It’s not quite so important for grassland apart the herb cover I think. The herb cover is important for most grassland. That would certainly affect your conservation assessment. But beyond that… I don’t think about groups of species in particular.”I8 Grasslands [Wales]*

The cover proportion of negative indicator species-groups can also be used as an indicator. For instance, in many grasslands a high proportion of grasses is seen as indicating lower habitat quality:

*“...looking at it from the negative side, we use grasses, proportion of grass cover I think quite successfully in the Common Standards, because it does seem to give a fairly good signature of nutrient status. So, if your total grass component is increasing, then the chances are that there is something going wrong in terms of the nutrient loading on the site. ... it didn’t work well with acid grasslands, because they are pretty grass-dominated by native species. So it didn’t work very well for those. But it works well for neutral grasslands, it works well for calcareous grasslands, for instance.” I10 Grasslands [England]*

High cover of graminoids (grasses, sedges, rushes and other narrow-leaved monocotyledonous species) is sometimes referred to as a negative condition indicator. The distinction between ‘grasses’ and ‘graminioids’ was explored in some interviews. The predominant view was that non-grass graminoids, particularly sedges, should not be generally included as negative indicators:

*“…when you’re thinking about desirable species, we tend to include them [*Carex *species]. Whether you call them actually forbs I don’t know. But I don’t think you would. But they are kind of desirable, usually.”I8 Grasslands [Wales]*

*“I don’t think I’ve ever come across* Carex *as being negative.” I13 Heaths [England]*

The percentage cover of dwarf shrubs appears to be a useful assessment tool for heathlands in all four countries, either as a measurement by themselves, or in proportion to other groups such as graminoids:

*“So on a heathland site we would look at overall dwarf shrub cover.” I9 Heaths [Northern Ireland]*

*“…one of the species compositions that should be looked at should be the cover dwarf shrubs and cover of graminoids. There’s quite a broad range for both of these, but … I suppose the ideal, if there is such a thing, would be about half of it covered by dwarf shrubs and half it covered with graminoids. It’s bit of an abstraction that, but within that – the targets within CSM for that – is that neither dwarf shrubs nor graminoids should be more than 75%,* so it gives you quite a wide range and it encompasses lots of different examples of the habitat.*” I4 Heaths [Scotland]*

Additionally, lichen and moss groups may be useful for habitat quality assessment in heathlands, for instance by providing information about habitat structure:

*“ The lichens are important, I think, mosses are important, and tells us a lot about the structure as well, so that’s important.” I5 Heaths [Wales]*

*“… if you used, certainly for the lichens, if you used... the* Cladonia *species, and split them into the* Cladina*, the bushy ones like* Cladonia arbuscula *and all that lot, and use those, plus* Cetraria *species, I think they are good indicators of good quality, and most of the rest of that you get up there are indicators that something else is going on, like those that are more tolerant of higher N-deposition.” I7 Heaths [Wales]*

Mosses, particularly the *Sphagnum* genus, are also useful for assessing the quality of wetland habitats. However, there is wide variation in the habitat requirements (e.g. on the oligotrophic-eutrophic axis) among mosses, and even among *Sphagna*. Observations of individual species may be necessary for gaining information regarding environmental conditions and habitat structure:

*“Well in bogs, we’re tending to use Sphagna as a group, but again, there’s a lot of variation in* Sphagna *from wet, sitting in ditches, and others from dry hummocks that are quite dry. So again, as a group we’re using that as a proxy measure for water content, and also consistency of water content.” I2 Wetlands [Scotland]*

## Topic 6: Reference communities

#### T6 a) Defining a reference community

Although there was recognition of the appeal of a reference community, there was a very strong consensus amongst the habitat specialists, irrespective of habitat specialisation or country, that it was a difficult and potentially risky task to attempt to define a reference community to compare a site against. This is due to the fact that habitats are naturally variable, both spatially and temporally, and a single reference point would not reflect the variation in high quality habitat, as illustrated below:

*“So I can see the attractiveness in all of this but it is quite difficult to define, and it’s almost saying, setting habitats in stone, saying ‘this is what it has to be’.” I4 Heaths, Wetlands, Grasslands [Scotland]*

*“Whether you could find a site that was representative of the range of variation… you would struggle... I would be concerned that it wouldn’t be representative of the range of variation that exists.” I3 Wetlands [Scotland]*

*“...you can’t define a best heathland – it’s a broad habitat and the composition, the species composition, changes with altitude and latitude.” I13 Heaths [England]*

Using an NVC community type as a reference community appeared to be a particularly problematic concept. Whilst the NVC is a useful tool for classifying habitats, it is considered to be too specific to act as a reference community, and wouldn’t capture the variation in habitats:

*“...the NVC is only a coat hanger on which to hang your vegetation, it doesn’t mean to say that just because you find something that is atypical it’s somehow of less intrinsic interest. In fact, you could argue in some cases it’s more interesting, so I think you have to be quite careful, that while the NVC is a useful tool for conservation assessment and communication between ecologists, it shouldn’t be viewed in that very rigid kind of way, and the recognition that it’s a continuum basically.” I10 Grassland [England]*

*“... it’s really difficult to say that, even recreating a habitat in a particular area it is difficult to say, well I’m aiming for this NVC class. I don’t think you can do it really, I think it’s very useful for us to classify vegetation, but it may not be useful for all the things.” I13 Heaths [England]*

The spatial scale of any reference community was also considered to be important, as explained in relation to heathlands:

*“I’m also not sure how you define a perfect heathland spatially, because heathland isn’t uniform, it’s patchy. So, I know one of the things we had issues with, I think Common Standards say there must be two species of ericoids. And, is that two species of ericoids within your sample, or is that two species of ericoids across the site? So if you are going to define it, you’re going to have to define it spatially as well – what you mean by ideal habitat. It’s not one patch.” I5 Heaths [Wales]*

The possibility of using a reference community based on predictions of future climates was considered risky due to limitations in climate model predictions:

*“… although I appreciate that climate change is happening, I don’t think our understanding is anywhere near the mark to start doing that. I think we could end up the creek without the paddle.” I2 Wetlands [Scotland]*

#### T6 b) Potential reference community definitions

Although a reference community was generally not a popular concept among the specialists, there was some consideration of how such a community might be chosen. For example, NVC communities may be an appropriate starting point, as long as flexibility was incorporated:

*“I think any kind of referencing needs a certain amount of flexibility to account for change within the communities as well. ... I think the NVC is probably the closest you’re going to get to have something that we all agree on that is relatively close to that single reference point, but around it there needs to be that grey area of a little bit of flexibility as well...” I2 Wetlands [Scotland]*

However, one reference community per habitat is also not likely to be sufficient, as indicated below:

*“So, I think you’d need more than one, you’d need a range or type-locations or type-states, that you could easily capture the geographical variation.” I6 Wetlands [Wales]*

CSM could be used as a starting point to devise a reference community. Such an approach has been attempted, but the outcome was not judged to be satisfactory:

*“It [a reference community] is something that we’ve sort of played around with a bit previously, because of the way CCW did the Common Standards Monitoring, we actually almost did it in that way. We had an idea of what a perfect piece of heathland should look like, and your quadrat should fit into that perfect piece of heathland. But I’ve not been convinced about it. I think there’s too much dissimilarity between different types of heathland. So... you can get a heathland that is very good, which doesn’t match up to your perfect heathland.” I5 Heaths [Wales]*

Using previous records of a site was considered to be a possible approach to defining a reference community, but such a reference would likely be very site-specific. Such records also seldom exist:

*“If you actually had old records for the site and could go back and compare, that would be very useful. I can’t think of any instances where you are likely to have good enough old records that you would compare with. That would be useful but impractical.” I1 Grasslands [Scotland]*

*“I don’t know whether our data really would be in a state where you could say well this site had these, and now they’ve gone. So looking back I’m pretty sure we couldn’t do it historically in the last 10 years.” I11b Wetlands [England]*

*“The one thing with wetlands is our recording has been very slow...we’ve got rough species lists, and that’s about as far as we’ve actually got.” I2 Wetlands*

Another specialist noted that for some habitats, extensive quadrat data have been gathered. Such data could potentially be used to help define a reference community:

*“We’ve got quite a big database of sites, so we’ve got hundreds of quadrats on MG5 for example, and we’ve got constancy values that give you the typical MG5 constancy for the whole of Wales, so that would be a starting point for this is what MG5 looks like in Wales, as a typical state.” I8 Grasslands [Wales]*
